# Triacylglycerol remodeling in *Physaria fendleri* indicates oil accumulation is dynamic and not a metabolic endpoint

**DOI:** 10.1093/plphys/kiab294

**Published:** 2021-06-24

**Authors:** Sajina Bhandari, Philip D. Bates

**Affiliations:** Institute of Biological Chemistry, Washington State University, Pullman, Washington 99164, USA

## Abstract

Oilseed plants accumulate triacylglycerol (TAG) up to 80% of seed weight with the TAG fatty acid composition determining its nutritional value or use in the biofuel or chemical industries. Two major pathways for production of diacylglycerol (DAG), the immediate precursor to TAG, have been identified in plants: de novo DAG synthesis and conversion of the membrane lipid phosphatidylcholine (PC) to DAG, with each pathway producing distinct TAG compositions. However, neither pathway fits with previous biochemical and transcriptomic results from developing *Physaria fendleri* seeds for accumulation of TAG containing >60% lesquerolic acid (an unusual 20 carbon hydroxylated fatty acid), which accumulates at only the *sn*-1 and *sn*-3 positions of TAG. Isotopic tracing of developing *P. fendleri* seed lipid metabolism identified that PC-derived DAG is utilized to initially produce TAG with only one lesquerolic acid. Subsequently a nonhydroxylated fatty acid is removed from TAG (transiently reproducing DAG) and a second lesquerolic acid is incorporated. Thus, a dynamic TAG remodeling process involving anabolic and catabolic reactions controls the final TAG fatty acid composition. Reinterpretation of *P. fendleri* transcriptomic data identified potential genes involved in TAG remodeling that could provide a new approach for oilseed engineering by altering oil fatty acid composition after initial TAG synthesis; and the comparison of current results to that of related Brassicaceae species in the literature suggests the possibility of TAG remodeling involved in incorporation of very long-chain fatty acids into the TAG *sn*-1 position in various plants.

## Teaser

In vivo isotopic tracing indicates *Physaria fendleri* incorporates industrially valuable unusual fatty acids into seed storage oil through a dynamic triacylglycerol remodeling process.

## Introduction

Oilseed plants accumulate triacylglycerol (TAG, oil) as the major form of seed carbon storage. TAG fatty acid composition determines its value for food, biofuels, or chemicals. Most food oils contain just five common fatty acid structures with 16–18 carbons and 0–3 double bonds. However, more than 450 different fatty acids with unusual structures are produced within the plant kingdom and can be valuable renewable feedstocks for the chemical industry (Dyer et al., 2008; Ohlrogge et al., 2018). Unfortunately, most of the unusual fatty acid accumulating plants are not suitable as crops. With few exceptions (e.g. unusual monounsaturated fatty acids; Nguyen et al., 2015), the bioengineering of unusual fatty acid production into model and crop plants has yet to achieve levels near that of native species, indicating we still do not fully understand the metabolic pathways required to accumulate select fatty acids in seed oils (Lee et al., 2015; Correa et al., 2020).

Hydroxy fatty acids (HFAs) are unusual fatty acids produced by various plants (Ohlrogge et al., 2018) and can be used in a wide variety of industrial products including lubricants, hydraulic fluids, biodegradable polyesters, polymers, coatings and paints, cosmetics, pharmaceuticals, perfumes, and biofuels (Mutlu and Meier, 2010; Carlsson et al., 2011). The major world source of HFAs is castor (*Ricinus communis*) oil that contains the 18-carbon HFA ricinoleic acid (18:1OH; [9Z,12R]-12-hydroxyoctadec-9-enoic acid). However, castor seeds also produce the extremely toxic protein ricin (Bradberry et al., 2003), which has led to the desire to engineer valuable HFAs into a more suitable crop (Carlsson et al., 2011). Most of the proof-of-concept bioengineering has been through transformation of the model plant *Arabidopsis thaliana* or the crop Camelina (*Camelina sativa*) with genes from castor or *Physaria fendleri* (formerly *Lesquerella fendleri*; Al-Shehbaz and O'Kane, 2002), which accumulates the 20-carbon HFA lesquerolic acid (20:1OH; (11Z,14R)-14-hydroxyeicos-11-enoic acid; Broun and Somerville, 1997; Smith et al., 2003; Lu et al., 2006; Dauk et al., 2007; Burgal et al., 2008; Dauk et al., 2009; van Erp et al., 2011; van Erp et al., 2015; Aryal and Lu, 2018; Lunn et al., 2019; Shockey et al., 2019). However, levels of HFAs in the transgenic seed oils (<40% in Arabidopsis [Lunn et al., 2019; Shockey et al., 2019], and <24% in Camelina [Aryal and Lu, 2018]) have not come close to the level in castor (80%–90%) or *P. fendleri* (>60%). In addition, inefficient accumulation of HFAs in TAG reduces total seed oil accumulation in transgenic Arabidopsis (Bates et al., 2014) and Camelina (Aryal and Lu, 2018). The limited ability to engineer designer fatty acid compositions of choice in transgenic plant oils indicates we still do not fully understand the mechanisms of plant TAG biosynthesis to be able to exploit these valuable natural resources (Bates, 2016).

**Figure 1:**
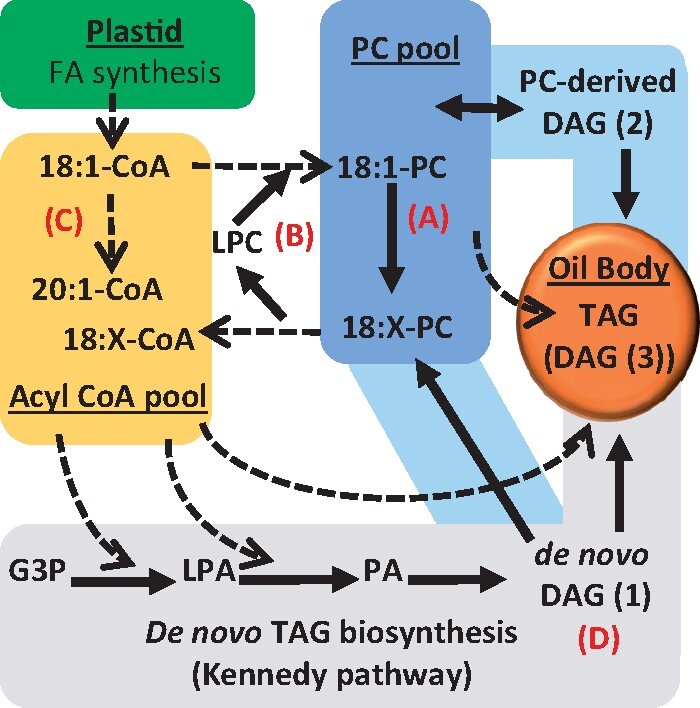
Plant lipid metabolic network of TAG assembly. TAG is synthesized from multiple substrate pools including at least three DAG pools and at least two acyl donors (acyl-CoA and PC). The path of acyl flux through the network into TAG ultimately determines the final oil fatty acid composition. Dotted lines indicate acyl group transfer, solid lines indicate flux of the glycerol backbone. A, Fatty acid modification on PC (e.g. desaturation, hydroxylation). B, Acyl editing cycle exchanging fatty acids between PC and acyl-CoA pools. C, Fatty acid elongation. D, De novo DAG(1) can be used directly for TAG synthesis as in the Kennedy pathway (gray shade), or can be used to produce PC and subsequently PC-derived DAG(2) prior to TAG synthesis (blue shade). A third pool of DAG(3) may be associated with the oil body and made up of both sources and can be used for TAG biosynthesis. G3P, glycerol-3-phosphate; LPA, lyso-phosphatidic acid; PA, phosphatidic acid.

Plant oil is synthesized from a metabolic network that overlaps with essential membrane lipid production (Figure 1), and has been extensively reviewed (Roughan and Slack, 1982; Ohlrogge and Browse, 1995; Bates and Browse, 2012; Li-Beisson et al., 2013; Allen et al., 2015; Chen et al., 2015; Bates, 2016). The relative flux of nascent fatty acids through various branches of the lipid metabolic network affects the fatty acid composition of the seed oil, including fatty acid synthesis in the plastid; fatty acid elongation (≥20C) as cytosolic acyl-CoAs; de novo diacylglycerol (DAG) assembly in the endoplasmic reticulum (ER); membrane lipid production from de novo DAG; fatty acid modification (e.g. desaturation, hydroxylation) on the membrane lipid phosphatidylcholine (PC); acyl editing exchange of fatty acids between PC and the acyl-CoA pool; PC-derived DAG production; TAG production from various DAG and acyl substrate pools; and acyltransferase substrate selectivity (Figure 1; Bates, 2016; Jeppson et al., 2019; Regmi et al., 2020). Control of acyl flux through the network into TAG is especially important for unusual fatty acids that can be detrimental to membrane structure and function (Millar et al., 2000). Inefficient flux of unusual fatty acids (produced transgenically) through membrane lipids prior to incorporation into TAG has been characterized as a major bottleneck to oilseed engineering (Napier, 2007; Bates and Browse, 2011; Bates et al., 2014; Yu et al., 2018). Yet, it is unclear how most plants control acyl flux to produce diverse TAG compositions (Bates, 2016).

Both castor and *P. fendleri* accumulate HFAs in seed oil, and both produce ricinoleic acid through hydroxylation of oleic acid attached to PC via fatty acid hydroxylases that are homologs of fatty acid desaturase (FAD2) enzymes (Bafor et al., 1991; Vandeloo et al., 1995; Reed et al., 1997; Broun et al., 1998; Shanklin and Cahoon, 1998). PC acyl editing liberates ricinoleoyl-CoA (Lager et al., 2013), which in castor is used for de novo synthesis of DAG containing two HFAs, and subsequently TAG containing three HFAs (3HFA-TAG) by the Kennedy pathway (Figure 1; Bafor et al., 1991). In *P. fendleri*, ricinoleoyl-CoA is first elongated to lesqueroyl-CoA before incorporation into TAG at just the *sn*-1/*sn*-3 positions (2HFA-TAG; Hayes and Kleiman, 1996; Reed et al., 1997; Moon et al., 2001). A Kennedy pathway of TAG production (as in castor) makes logical sense to keep lesquerolic acid out of membrane lipids in *P. fendleri*; however, multiple pieces of evidence challenge this assumption. First, genetic analysis indicates that *P. fendleri* is closely related to fellow Brassicaceae species Arabidopsis and Camelina (Horn et al., 2016), both of which utilize a PC-derived DAG pathway of TAG synthesis as demonstrated by in vivo isotopic tracing of lipid metabolism (Bates and Browse, 2011; Yang et al., 2017). Second, PC:DAG cholinephosphotransferase (*PfePDCT*; a homolog of Arabidopsis reduced oleate desaturase1 [*AtROD1*], and a key producer of PC-derived DAG in Arabidopsis; Lu et al., 2009) is highly expressed in developing *P. fendleri* seeds (Kim and Chen, 2015; Horn et al., 2016). Third, the evaluation of protein sequences that likely evolved to utilize HFAs in *P. fendleri* as compared to eight non-HFA accumulating Brassicaceae species indicated that only the last step of the Kennedy pathway (acyl CoA:DAG acyltransferase, *PfeDGAT1* or *PfeDGAT2*) but not the initial step (glycerol-3-phosphate acyltransferase, *PfeGPAT9*; Shockey et al., 2016) demonstrated significant sequence divergence (Horn et al., 2016), suggesting the lack of a HFA selective GPAT for a direct Kennedy pathway of 2HFA-TAG production. Together, this previous genetic analysis is suggestive of a PC-derived DAG pathway of TAG biosynthesis; however, analysis of *P. fendleri* lipids does not fit with this assumption either. First, the combination of hydroxylase activity on PC and PC-derived DAG production should lead to ricinoleic acid accumulation predominantly at the *sn*-2 position of TAG (as it does in transgenic Arabidopsis expressing the castor hydroxylase; van Erp et al., 2011; Hu et al., 2012), yet this does not occur in *P. fendleri* (Chen et al., 2020). Second, PC containing lesquerolic acid (as an intermediate to PC-derived DAG containing lesquerolic acid for TAG synthesis) has not been found in *P. fendleri* (Chen et al., 2011). Thus, current genetic and biochemical evidence appear to be conflicting on how *P. fendleri* could utilize a PC-derived DAG pathway of TAG biosynthesis to specifically accumulate HFAs at only the *sn-1/sn-3* positions of 2HFA-TAG.

Therefore, to better understand the pathway of TAG biosynthesis, we traced in vivo lipid metabolism with isotopic labeled substrates and demonstrated that *P. fendleri* produces DAG without HFAs (0HFA-DAG) from PC for TAG synthesis and subsequently utilizes a previously unidentified TAG remodeling mechanism that removes common fatty acids from TAG and incorporates lesquerolic acid. Reinterpretation of previous transcriptomic results indicates potential biotechnical tools that may allow designer seed oil compositions by changing the seed oil fatty acid composition after initial TAG synthesis.

## Results

### TAG containing 0, 1, or 2 HFAs accumulate at different rates in developing seeds

**Figure 2:**
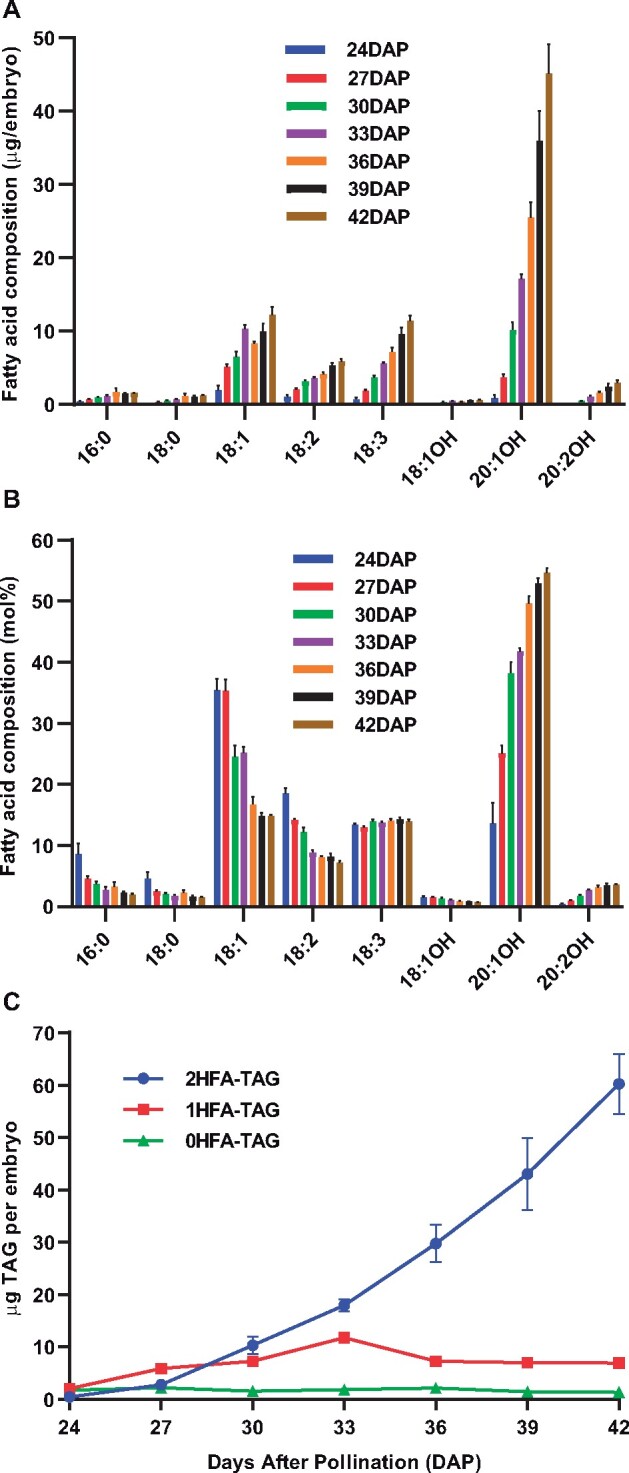
Fatty acid composition and TAG accumulation across *P. fendleri* embryo development. A, Fatty acid composition as µg per embryo. B, Fatty acid composition as mole percentage. C, TAG accumulation per embryo. Data are mean ± sem of five replicates for each stage except for 24 DAP, three replicates.

Many Brassicaceae species of the *Lesquerella* and *Physaria* genera accumulate HFAs in seed TAG, or as estolides of TAG where additional HFAs are esterified to the mid chain hydroxyls producing molecules with four to five fatty acids (Hayes et al., 1995; Ohlrogge et al., 2018). *Physaria* *fendleri* seeds mature over ∼50 d (Reed et al., 1997; Chen et al., 2009) and we confirmed previous reports that lesquerolic acid (20:1OH) accumulates over seed development predominantly in 2HFA-TAG (Figure 2), and not as TAG estolides (Hayes et al., 1995; Hayes and Kleiman, 1996; Lin and Chen, 2013, 2014; Lin et al., 2015). The identity of each HFA-containing lipid species separated by thin-layer chromatography (TLC) as a TAG and not as an estolide was confirmed by the fatty acid composition where 2HFA-TAG and 1HFA-TAG (HFA at *sn*-1 or *sn*-3) contained approximately 2/3 and 1/3 HFA, respectively (Supplemental Figure S1, A and B). In addition, we confirmed previous reports that lesquerolic acid is not detected in PC (Supplemental Figure S1D; Chen et al., 2011). Though HFAs accumulate in lipids at early stages (Figure 2, A and B; Cocuron et al., 2014) 0HFA-TAG and 1HFA-TAG are initially produced more than 2HFA-TAG (Figure 2C). 1HFA-TAG continues to accumulate until 33 d after pollination (DAP) and then levels decrease. However, 2HFA-TAG accumulates rapidly after 27 DAP, and is the major TAG species accumulated by and beyond 30 DAP (Figure 2C). To better understand the pathway of HFA incorporation into 2HFA-TAG, developing *P. fendleri* embryos at 30 DAP were utilized for both rapid pulse and long-term pulse-chase isotopic tracing of newly synthesized fatty acids and glycerol backbones through lipid metabolism (Allen et al., 2015).

### Tracing of newly synthesized fatty acid flux through the glycerolipid metabolic network

**Figure 3:**
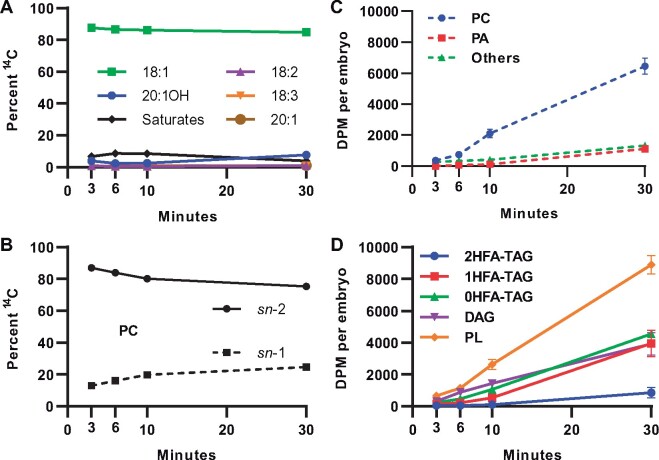
^14^C-lipid accumulation from continuous [^14^C]acetate labeling of developing *P. fendleri* embryos. A, Total lipid radiolabeled fatty acid composition. B, Stereochemistry of labeled fatty acid accumulation in PC. C, Accumulation of radiolabeled fatty acids in PLs. D, Accumulation of radiolabeled fatty acids in neutral lipids and total PLs. Each data point is mean ± sem of three individual labeling replicates. Each time point replicate contained 10 embryos at 30 DAP, 120 total embryos for the time course.

Short time point continuous [^14^C]acetate metabolic labeling from 3 to 30 min was utilized to determine the initial paths of newly synthesized fatty acids fluxes through the lipid biosynthetic network into TAG (Allen et al., 2015; Figure 3). Total ^14^C incorporation into lipids was linear, indicating continual lipid biosynthesis in the developing embryos over the metabolic labeling time course (Supplemental Figure S2). Nascent ^14^C-labeled fatty acids (mostly composed of oleic acid; Figure 3A) were initially incorporated into the *sn*-2 position of PC (Figure 3, B and C) faster than incorporation into any TAG molecular species (Figure 3D). This result is consistent with incorporation of nascent fatty acids first into PC as they exit the plastid through acyl editing rather than directly into de novo glycerolipid synthesis (Bates et al., 2009, 2012; Yang et al., 2017; Karki et al., 2019; Figure 1). The initial incorporation of newly synthesized fatty acids into TAG species was mostly into 0HFA-TAG, followed by 1HFA-TAG, with very little into 2HFA-TAG (Figure 3D). Despite lesquerolic acid representing ∼9% of labeled fatty acids at 30 min (Figure 3A), 2HFA-TAG represented ∼3% of total labeled lipids (Figure 3D).

**Figure 4:**
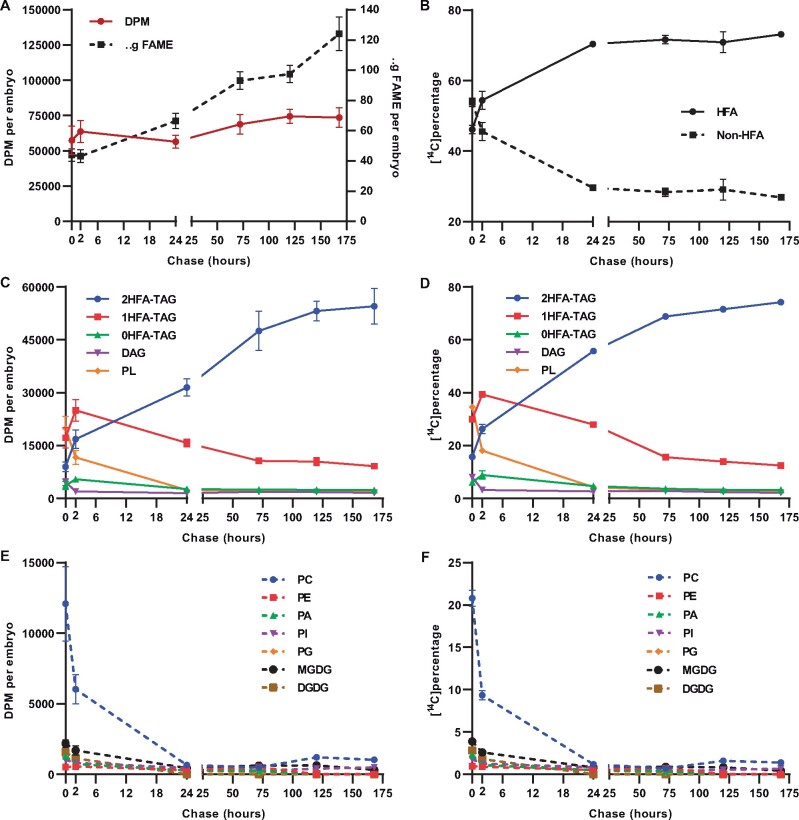
[^14^C]acetate pulse-chase labeling of developing *P. fendleri* embryos. One hour pulse with 30 DAP embryos followed by chase time points of 2, 24, 72, 120, and 168 h. A, Total lipid accumulation (dotted line) and radioactivity incorporated in total lipids (solid line). B, Proportion of total ^14^C-HFAs and ^14^C-non-HFAs. ^14^C-fatty acid accumulation into neutral lipids and total PLs measured as (C) DPM per embryo and (D) ^14^C Percentage. ^14^C-fatty acid accumulation into individual PLs measured as (E) DPM per embryo (F) ^14^C Percentage. Data are mean ± sem of three individual labeling replicates of 10 embryos for each time point replicate, 180 total embryos for the time course.

To further elucidate the *P. fendleri* TAG assembly pathway over a longer period, a second experiment utilized a 1 h [^14^C]acetate pulse followed by a 168 h chase (Figure 4). During the chase, the level of total ^14^C-labeled fatty acids remained approximately constant but both total lipid mass and HFA content increased (Figure 4A;  Supplemental Figure S3), indicating continual lipid biosynthesis and no net fatty acid degradation during the chase. By 24 h, the ^14^C-HFA content stabilizes at ∼71% of total labeled fatty acids (Figure 4B), indicating little to no additional ^14^C-HFA synthesis during the next 6 d of chase. *Physaria* *fendleri* accumulates HFAs up to ∼60% of seed fatty acids (Figure 2), the higher proportion of labeled HFA to non-HFA is likely due to the higher specific activity of labeling during cytosolic fatty acid elongation than de novo fatty acid synthesis in the plastid as has been reported in other species that accumulate very long-chain fatty acids (≥20 carbons; Pollard and Stumpf, 1980; Bao et al., 1998; Bates et al., 2012; Pollard et al., 2015).

The incorporation of ^14^C-fatty acids into individual lipids was quantified as both total radioactivity per lipid per embryo, and as a percentage of total labeled lipids (Figure 4, C–F). Similar to the short time course labeling (Figure 3D), the polar lipids (PLs) comprised mostly PC that contained more labeled nascent fatty acids than any TAG species at the end of pulse (Figure 4, C–F), even considering the higher specific activity of the HFAs in TAGs. Over the first 2 h of the chase, radioactivity in PC and 0HFA-DAG decreased and labeled fatty acids accumulated predominantly in 1HFA-TAG followed by 2HFA-TAG and 0HFA-TAG (Figure 4, C–F). However, over the prolonged chase, the amount of labeled fatty acids in both 1HFA-TAG and 0HFA-TAG decreased while that of 2HFA-TAG increased (Figure 4, C and D). The decrease in PC labeling over the first 24 h (Figure 4, E and F) corresponded with the accumulation of labeled HFAs as the major labeled fatty acids (Figure 4B), consistent with the hydroxylation of fatty acids in PC but their accumulation in TAG species. The level of labeled HFAs increased only slightly after 24 h (Figure 4B), indicating that the large increase in ^14^C-2HFA-TAG through the remaining time course was predominantly not due to ^14^C-HFA synthesis but primarily due to the transfer of labeled fatty acids from 1HFA-TAG to 2HFA-TAG (Figure 4, C and D).

**Figure 5:**
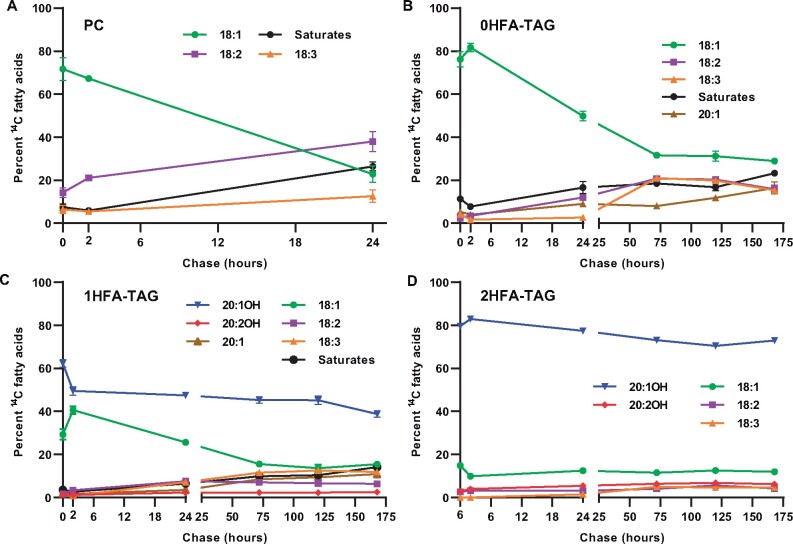
Relative ^14^C-fatty acid accumulation in different lipid species during [^14^C]acetate pulse-chase labeling of developing *P. fendleri* embryos. A, PC. B, 0HFA-TAG. C, 1HFA-TAG. D, 2HFA-TAG. Data are mean ± sem of three individual labeling replicates of 10 embryos for each time point replicate, 180 total embryos for the time course.

The changing composition of ^14^C-non-HFAs over the time course also indicates continual transfer of acyl groups between lipid species (Figure 5). In PC, the amount of ^14^C-18:1 incorporated during the pulse rapidly decreased over the first 24 h of chase and the amount of polyunsaturated fatty acids (PUFAs, 18:2, 18:3) increased (Figure 5A) due to desaturation and removal of ^14^C-fatty acids from PC (Figure 4, E and F). Over the first 2 h of chase as total levels of labeled 1HFA-TAG and 0HFA-TAG increased (Figure 4, C and D) the ^14^C-18:1 content in 1HFA-TAG and 0HFA-TAG also increased (Figure 5, B and C). However, during the remainder of the chase as total levels of labeled 1HFA-TAG and 0HFA-TAG decreased (Figure 4, C and D; 2–168 h), the ^14^C-18:1 content decreased and PUFA increased (Figure 5, B and C). ^14^C-PUFA even increased in 2HFA-TAG over the chase (Figure 5D). PC is the site for fatty acid desaturation and hydroxylation (Bates, 2016), and 18:1 released from PC through acyl editing is the main substrate for elongation to 20:1 (Bao et al., 1998; Bates et al., 2012). Therefore, the increase in labeled PUFA and 20:1 content of TAG species over the chase requires ^14^C-acyl flux through PC prior to the incorporation in TAG. Interestingly, by 24 h of chase, most radioactivity in PC has been depleted (Figure 4, E and F), and PC-18:1 and PC-PUFA represent just 0.27% and 0.60% of the total ^14^C-fatty acids, respectively (Supplemental Table S1). However, from 24 to 168 h, the percentage of total ^14^C-fatty acids as PUFA and 20:1 in TAG species increased 2.51% and 0.48%, respectively (Supplemental Table S1). In addition, total ^14^C-HFAs increased 3% over the same time (Figure 4B). Thus, the changes in each TAG species labeled FA composition from 24 to 168 h are over six-fold higher than the substrates available in PC at 24 h of chase. These results suggest that ^14^C-18:1 is likely removed from 1HFA-TAG and 0HFA-TAG, and reincorporated into PC for desaturation, hydroxylation, and eventual elongation. Together the quantitative changes in labeled fatty acid accumulation and composition (Figures 4, 5; Supplemental Table S1) in different lipids over the time course indicates a precursor–product relationship between where nascent fatty acids are first incorporated into PC, subsequently incorporated into 1HFA-TAG and 0HFA-TAG and then removed, with some reincorporation into PC for desaturation/hydroxylation, before final accumulation predominantly in 2HFA-TAG.

### Tracing of glycerol backbone flux through glycerolipid assembly

**Figure 6:**
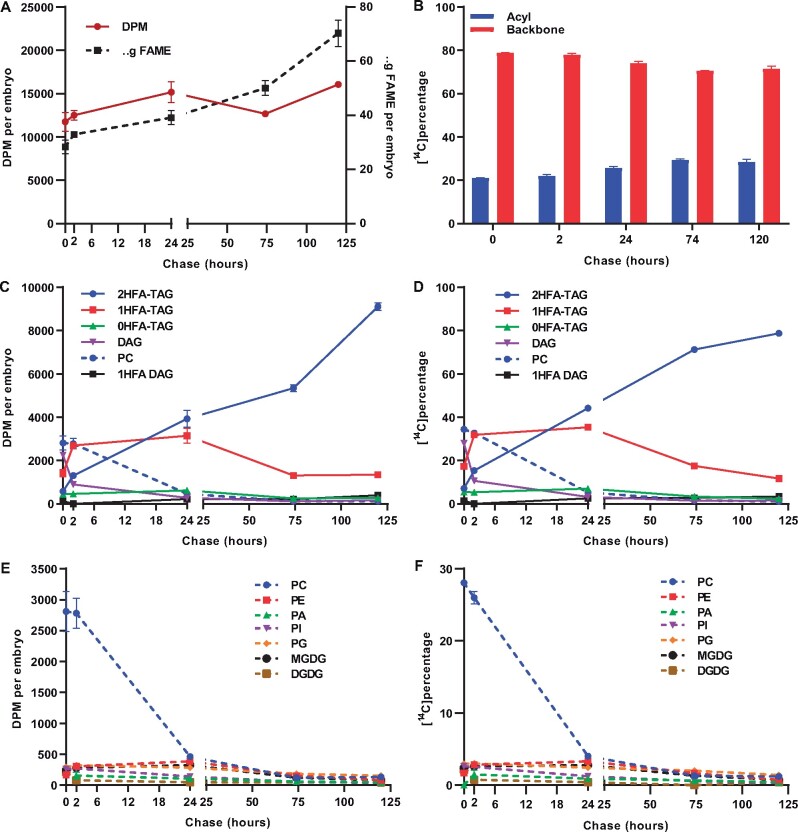
[^14^C]glycerol pulse-chase labeling of developing *P. fendleri* embryos. One hour pulse followed by chase time points of 0, 2, 24, 74, and 120 h. A, Total lipid accumulation (dotted line) and radioactivity incorporated in total lipid (solid line). B, ^14^C incorporation into the acyl and backbone fraction of total lipids. [^14^C]glycerol incorporation into backbones of neutral lipids and PC measured as (C) DPM per embryo (D) ^14^C Percentage. [^14^C]glycerol incorporation into backbones of individual PLs measured as (E) DPM per embryo (F) ^14^C percentage. Data are mean ± sem of three individual labeling replicates of 10 embryos for each time point replicate, 150 total embryos for the time course.

The [^14^C]acetate metabolic labeling indicated that nascent ^14^C-labeled fatty acids initially incorporated into PC, 0HFA-DAG, 0HFA-TAG, and 1HFA-TAG were removed and incorporated into the major TAG species 2HFA-TAG (Figure 4) predominantly as ^14^C-HFAs (Figures 4B and 5D). However, it is unclear if only the acyl chains are transferred between glycerolipid species, or if the glycerol backbone also moves between each lipid. [^14^C]glycerol pulse-chase labeling was performed to track the flux of glycerol through the lipid metabolic network (Figure 6). During the chase, the level of total ^14^C-labeled lipids remained approximately constant but both total lipid mass and HFA content increased (Figure 6A;  Supplemental Figure S4), indicating continual lipid biosynthesis and no net fatty acid degradation during the 120-h chase period. Radioactivity from [^14^C]glycerol accumulated approximately 70%–80% in the glycerol backbone fraction of total lipids and approximately 20%–30% in the acyl chains (Figure 6B). Therefore, to trace the flux of just the labeled glycerol backbone between individual lipids over the time course, each lipid was collected, transmethylated, and the radioactivity in just the glycerol backbone fraction of each lipid was quantified as both total radioactivity per embryo and the percent of total backbone labeled lipids (Figure 6, C–F).

After an hour of pulse, the backbone labeling of 0HFA-DAG and PC was higher than all TAG species (Figure 6, C and D), and both decreased during the chase. The rapid 0HFA-DAG labeling, the 0HFA-DAG to PC precursor–product relationship, the very limited labeling of 1HFA-DAG, and the long lag in 2HFA-TAG labeling suggests a lack of a direct Kennedy pathway of 2HFA-TAG production, and that de novo PC production utilizes DAG without HFAs. In addition, the rapid flux of [^14^C]glycerol into PC as compared to other PLs (Figure 6, E and F), and combined with the decrease in labeled PC and increase in labeled TAGs over the chase (Figure 6, C and D) supports that the glycerol backbone of the membrane lipid PC is an intermediate to the biosynthesis of TAGs consistent with the PC-derived DAG pathway of TAG biosynthesis found in related Brassicaceae species: Arabidopsis and Camelina (Bates and Browse, 2011; Yang et al., 2017). The [^14^C]glycerol backbone labeling of 1HFA-TAG increased during the first 2 h of chase concomitant with decreases in PC and 0HFA-DAG, suggesting PC-derived DAG without HFAs is acylated with a HFA to produce 1HFA-TAG. The leveling off of 1HFA-TAG labeling from 2 to 24 h concomitant with a large decrease in PC and an increase in 2HFA-TAG is representative of combined 1HFA-TAG synthesis (from PC) and 1HFA-TAG turnover providing substrate for 2HFA-TAG accumulation (Figure 6, C and D). Finally, the decrease in 1HFA-TAG concomitant with a similar increase in 2HFA-TAG from 24 to 120 h is consistent with 1HFA-TAG providing the [^14^C]glycerol backbone for 2HFA-TAG production (Figure 6, C and D). A similar but more subdued pattern is also evident for 0HFA-TAG (Figure 6, C and D). Therefore, the metabolic tracing of *P. fendleri* lipid metabolism with both [^14^C]acetate and [^14^C]glycerol indicated that the acyl chains and glycerol backbones of PC and 0HFA-DAG transiently accumulate in 0HFA-TAG and 1HFA-TAG prior to final accumulation in 2HFA-TAG.

## Discussion

### *Physaria fendleri* utilizes PC-derived DAG and TAG remodeling to accumulate 2HFA-TAG

Previous research in a variety of oilseed plants has indicated two major ways to produce the DAG substrate for TAG synthesis, de novo DAG synthesis through the Kennedy pathway and PC-derived DAG production (Bates, 2016). However, both previous genetic and biochemical analyses of *P. fendleri* TAG production do not fit with either pathway as a mechanism to accumulate lesquerolic acid at the *sn*-1/3 positions of 2HFA-TAG as the major molecular species in *P. fendleri* oil. In addition, TAG produced during the oil accumulation phase of seed development is typically considered a metabolic endpoint. The results presented herein utilizing in vivo isotopic tracing of lipid metabolism in developing *P. fendleri* embryos provide two major conclusions that clarify the process of 2HFA-TAG biosynthesis. First, *P. fendleri* predominantly utilizes the PC-derived DAG pool for initial TAG biosynthesis as indicated by the strong PC-TAG precursor–product relationships for both [^14^C]fatty acids and [^14^C]glycerol flux into lipids (Figures 4, 6). Second, the fatty acid composition of initially synthesized TAGs (mostly 1HFA-TAG) changes over time to finally accumulate 2HFA-TAG (Figures 4–6), indicating initially synthesized TAGs are not a metabolic endpoint but a dynamic metabolically active pool.

Seven pieces of evidence suggest that the major pathway of 2HFA-TAG accumulation is through TAG remodeling. TAG remodeling is defined as a change in TAG molecular species composition without complete TAG degradation and resynthesis. (1) During the [^14^C]acetate continuous pulse and pulse-chase experiments, lipid mass accumulation was consistent with 2HFA-TAG accumulation; however, labeled HFAs and non-HFAs are initially incorporated into 0HFA-TAG and 1HFA-TAG faster than 2HFA-TAG (Figure 3D;  Supplemental Figure S3; Figure 4, A, C, and D; 0–2 h). (2) Labeled 2HFA-TAG continues to increase during the [^14^C]acetate pulse-chase past the point where ^14^C-HFA production has mostly stopped (Figure 4, B–D; 24–168 h), indicating the ^14^C-HFAs are transferred to 2HFA-TAG from other lipid species. (3) Increases in acyl labeled 2HFA-TAG coincide with decreases in acyl labeled 0HFA-TAG and 1HFA-TAG species suggesting a precursor–product relationship (Figure 4, C and D). (4) The increases in PUFA and 20:1 in TAG species during the prolonged chase is consistent with TAG remodeling by ^14^C-18:1 removal from 0HFA-TAG and 1HFA-TAG, and entry into the PC:acyl-CoA acyl editing cycle for subsequent desaturation or elongation, prior to final accumulation in TAG species (Figure 5 and Supplemental Table S1). (5) During the [^14^C]glycerol pulse-chase experiment, lipid mass accumulation was consistent with accumulation of mostly 2HFA-TAG over the time course (Figure 6A, C and D; Supplemental Figure S4); however, the backbone labeling of 1HFA-TAG exceeded that of 2HFA-TAG until 24 h of chase (Figure 6, C and D), indicating initial HFA-TAG biosynthesis from PC-derived DAG favors 1HFA-TAG over 2HFA-TAG. (6) During the [^14^C]glycerol pulse-chase, 2HFA-TAG continues to accumulate past the point when the precursor pools of the PC-derived DAG pathway have been mostly depleted of labeled species, and this 2HFA-TAG backbone labeling coincides with decreases in backbone labeled 0HFA-TAG and 1HFA-TAG (Figure 6, C–F; 24–120 h), indicating a strong precursor–product relationship of the glycerol backbone moving from 0HFA-TAG and 1HFA-TAG species to 2HFA-TAG. (7) The accumulation of glycerol-labeled 2HFA-TAG is likely not from complete catabolism of 0/1HFA-TAG species liberating glycerol and fatty acids with subsequent 2HFA-TAG synthesis. If [^14^C]glycerol was liberated from TAG species initially labeled during the pulse and then re-entered TAG biosynthesis during the chase the subsequent [^14^C]glycerol labeling would mimic restarting the pulse and the proportion of newly synthesized TAG molecular species produced should match that produced at the end of the pulse, and thus little to no 2HFA-TAG would be labeled (Figure 6, 0–2 h). In addition, labeled intermediates of complete TAG catabolism (e.g. monoacylglycerol [MAG]) were not detected. Taken together these results indicate that PC-derived DAG is utilized to predominantly produce 1HFA-TAG, but this is not the final TAG composition, and that the major TAG species which accumulate (2HFA-TAG, Figure 2) is produced predominantly through remodeling of 1HFA-TAG to incorporate a second HFA.

### Relative flux and cellular organization of TAG assembly pathways

The pattern and dynamics of the individual lipid [^14^C]glycerol labeling curves reveal several important aspects of the organization and relative flux through various branches of the *P. fendleri* lipid metabolic network (Figure 1). First, we can estimate the maximal possible amount of direct Kennedy pathway production of 2HFA-TAG versus TAG production utilizing PC-derived DAG (Figure 1). At the end of the pulse, 2HFA-TAG represents ∼7.1% of [^14^C]glycerol backbone labeled lipids (Figure 6D), and of the remaining labeled lipids only 1HFA-DAG at 1.5% could be a direct Kennedy pathway intermediate to produce 2HFA-TAG during the chase. Therefore, assuming both of these lipids are only assembled by the Kennedy pathway, a maximum of 8.6% of total glycerolipid assembly would be utilized for direct Kennedy pathway 2HFA-TAG production, with the remainder of Kennedy pathway glycerolipid assembly predominantly used for PC synthesis and subsequent TAG synthesis from PC-derived DAG (Figures 1 and 6, D). Similar ratios of Kennedy pathway versus TAG synthesis from PC-derived DAG have also been indicated in other Brassicaceae species (Arabidopsis and Camelina) by measuring the initial rate of TAG and PC synthesis from short time point (e.g. <10 min) continuous [^14^C]glycerol labeling studies (Bates and Browse, 2011; Yang et al., 2017). However, it is also possible that during the long 1-h pulse (Figure 6D), some of the labeled 2HFA-TAG may have come from initially labeled PC-derived DAG and TAG remodeling. Thus, the level of direct Kennedy pathway production of 2HFA-TAG in *P. fendleri* may be <8.6%.

Second, the different synthesis and turnover rates of [^14^C]glycerol-labeled 0HFA-DAG, PC, and 1HFA-TAG indicate both the order of intermediate metabolic reactions and differences in intermediate pool sizes leading to 2HFA-TAG accumulation (Figures 6 C, D and Supplemental Figure S6). In a multistep metabolic pathway with constant overall flux, the reaction rate of each individual step is related to the intermediate pool size in a first-order reaction, *k*[*S*] (Segel, 1976). During the midstage of *P. fendleri* oil accumulation used for metabolic labeling (30–36 DAP; Figure 2), 2HFA-TAG accumulates at a rate of 4.75 nmol/embryo/day, and the intermediates pools of PC and 1HFA-TAG are approximately constant at 0.82 and 12.13 nmol embryo^−1^, respectively (Supplemental Figure S5). Therefore, assuming total PC and 1HFA-TAG are intermediates to 2HFA-TAG synthesis, then the turnover rates for PC and 1HFA-TAG are 5.74 and 0.39 nmol embryo^−1^ day^−1^, respectively, to achieve constant flux. This estimate of relative flux through the PC and 1HFA-TAG pools is consistent with their different rates of [^14^C]glycerol labeling and turnover (Figure 6). When such a pathway is traced with pulse-chase metabolic labeling the maximal fraction of labeled versus unlabeled molecules in an individual metabolite pool only occurs after the precursor pools have reached their maximal fraction of labeling (Segel, 1976; Allen et al., 2015). Once maximal labeling is reached the efflux of labeled molecules from that pool will be reflected in an exponential decay curve (Sin et al., 2016). The exponential decay is due to the decreasing chance a labeled molecule will be reacted on by the next enzyme in the sequence over that of an unlabeled molecule, and the half-life of labeled molecules in each metabolite pool is therefore dependent on pool size. In Figure 6D, the rapid decrease in [^14^C]glycerol-labeled 0HFA-DAG can be fit to an exponential decay curve starting at *t *= 0 and with a half-life of 1.3 h (Supplemental Figure S6), indicating a small pool that is rapidly turned over with little to no additional pool filling during the chase. Similar studies have also suggested that de novo DAG is a small and rapidly turned over pool in soybeans (*Glycine max*; Bates et al., 2009). The small change in [^14^C]glycerol-labeled PC during the first 2 h of chase (Figure 6D) suggests that maximal PC pool labeling is between 0 and 2 h. PC labeling fits an exponential decay curve starting at 2 h of chase with a half-life of 7.3 h consistent with PC as a product of 0HFA-DAG, and representing a larger metabolite pool (Supplemental Figure S6). 1HFA-TAG reaches maximal labeling between 2 and 24 h of chase (Figure 6D) and can be fitted to an exponential decay curve starting at 24 h (Supplemental Figure S6) consistent with 1HFA-TAG as a downstream product of both 0HFA-DAG and PC, and the half-life of 32.5 h indicates 1HFA-TAG is an intermediate to final TAG accumulation with a much larger pool size than either 0HFA-DAG and PC, consistent with the mass measurements (Figure 2; Supplemental Figure S5). Therefore, the timing of when each labeled lipid fits to an exponential decay curve supports that 0HFA-DAG, PC, and 1HFA-TAG are all sequential intermediates toward accumulation of the final product 2HFA-TAG, and the increasing half-life of labeled lipids in each pool is supported by differences in pool size (Supplemental Figure S5).

Third, the 2HFA-TAG labeling does not display an exponential decay indicating it is the final metabolic product as expected by the lipid accumulation in Figure 2. However, the labeling of end-products typically lags until all precursor pools have filled. Despite less label in 2HFA-TAG than PC, 0HFA-DAG, or 1HFA-TAG at the end of the pulse (Figure 6D), the initial rapid labeling of 2HFA-TAG (Figure 6; 0–2 h) appears to occur before maximal labeling of 1HFA-TAG. This observation can be explained by the cellular organization of oil biosynthesis and accumulation. TAG is produced in the ER but accumulates in oil bodies; therefore, it is likely that a small pool of initially produced 1HFA-TAG can be quickly remodeled to 2HFA-TAG; however, most ^14^C-1HFA-TAG diffuses into the much larger pool of unlabeled 1HFA-TAG in the oil body that produces the long half-life of labeled 1HFA-TAG turnover (Figure 6D;  Supplemental Figures S5 and S6). Finally, the turnover of 1HFA-TAG in the oil body is an important observation that supports the current view that developing seed oil bodies are not just TAG storage compartments but are metabolically active organelles (Gao and Goodman, 2015; Pyc et al., 2017), here we demonstrate that oil body metabolism now also includes TAG remodeling.

### Model of 2HFA-TAG biosynthesis through TAG remodeling

The TAG remodeling demonstrated by our isotopic tracing of *P. fendleri* TAG biosynthesis could be explained by multiple possible mechanisms. First, a simple explanation is that 1HFA-TAG is directly hydroxylated to 2HFA-TAG. However, hydroxylases that act on TAG have yet to be identified, and previous work suggests the *P. fendleri* hydroxylase is a FAD2 homolog that acts on PC (Broun et al., 1998, 1998) thus this possibility is not considered further. Second, transacylases have been suggested to exchange FAs between DAG and TAG species. Castor 3HFA-TAG was demonstrated to transfer acyl groups to 0/1HFA-DAG producing 1HFA- and 2HFA-TAG (Mancha and Stymne, 1997), and the forward and reverse activity of a DAG:DAG transacylase producing TAG and MAG was suggested to be involved. However, DAG:DAG transacylase enzymes have not been identified and may actually be side reactions of phospholipid:diacylglycerol acyltransferase (PDAT; Ghosal et al., 2007; Yoon et al., 2012). Similar TAG:DAG or DAG:DAG transacylase mechanisms in *P. fendleri* are unlikely for three reasons: (1) unlike castor where the major TAG species (3HFA-TAG) is the acyl donor, the TAG remodeling in *P. fendleri* involves transfer of fatty acids and glycerol from minor TAG species (0/1HFA-TAG) to accumulate in the major TAG species (2HFA-TAG) thus this reaction is against a substrate gradient (Figures 4 and 6); (2) the transfer of a HFA from 1HFA-TAG to 1HFA-DAG producing 2HFA-TAG does not support the movement of the [^14^C]glycerol backbone from 1HFA-TAG to 2HFA-TAG, in addition there is hardly any [^14^C]glycerol-labeled 1HFA-DAG after the pulse that could lead to labeled 2HFA-TAG by transacylation with 1HFA-TAG (Figure 6); (3) DAG:DAG transacylation produces MAG, we did not detect any labeled MAG with either [^14^C]acetate or [^14^C]glycerol suggesting both *sn*-1/3 fatty acids of 0HFA-TAG are not removed at the same time. Thus, a DAG:DAG transacylase (Mancha and Stymne, 1997) or a MAG acyltransferase to produce DAG is likely not a required part of the TAG remodeling process.

**Figure 7:**
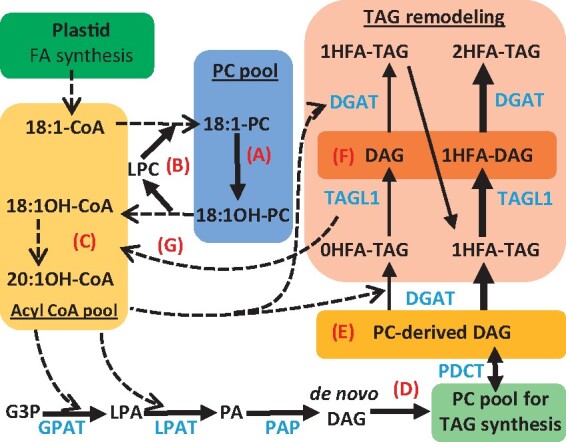
Metabolic pathway for incorporation of HFA into *P. fendleri* TAG. Solid lines indicate glycerol backbone transfer where thicker lines represent larger flux. Dotted lines indicate acyl group transfer. A, Hydroxylation of oleic acid (18:1) to ricinoleic acid (18:1OH) in PC. B, Acyl editing cycle to transfer 18:1OH to the acyl CoA pool. C, Elongation of 18:1OH to lesquorelic acid (20:1OH). D, De novo DAG is predominantly utilized for PC synthesis. E, Initial TAG is predominantly produced from PC-derived DAG (*sn*-1/2) not containing an HFA. F, TAG remodeling initially removes non-HFAs from TAG generating *sn*-1/2 or *sn*-2/3 DAG from 0HFA-TAG, and *sn*-2/3 DAG from 1HFA-TAG remodeling. G, Acyl groups removed by TAG remodeling are reincorporated into the acyl-CoA pool and may be further modified on PC and elongated. Enzyme abbreviations: DGAT, diacylglycerol acyltansferase; GPAT, glycerol-3-phosphate acyltransferase; LPAT, lyso-phosphatidic acid acyltransferase; PAP, phosphatidic acid phosphatase; TAGL1, TAGLipase like-1. Substrate abbreviations: G3P, glycerol-3-phosphate; PA, phosphatidic acid.

A third, and mostly likely possibility is a TAG lipase-based mechanism to produce 2HFA-TAG from PC-derived DAG and TAG remodeling as demonstrated in Figure 7. Initially, 1HFA-TAG is produced from 0HFA-DAG (*sn*-1/2) derived from PC, indicating the HFA is at the *sn*-3 position. To produce 2HFA-TAG, the s*n*-1 FA is removed by a lipase (producing *sn*-2-acyl-3-HFA-DAG) and replaced with a HFA by DGAT. As lesquerolic acid is not found in PC, the forward reaction of PDAT does not likely produce 2HFA-TAG. Conversion of the low amounts of 0HFA-TAG initially synthesized (Figures 2 and 6) to 2HFA-TAG will require remodeling at both the *sn*-1/3 positions (Figure 7). The very low levels of labeled 1HFA-DAG measured (Figure 6) suggest the possibility of channeled lipase/DGAT reactions that limit the accumulation of this intermediate. To accumulate 2HFA-TAG rather than regenerate 1HFA-TAG, the DGAT involved is likely selective for lesquerolyl-CoA, acyl-CoA selective DGATs are common in oilseed plants (Burgal et al., 2008; Jeppson et al., 2019; Shockey et al., 2019). The hypothesized lipase may or may not need to be selective for removal of non-HFAs. A fatty acid selective lipase could cleave non-HFAs from 0/1HFA-TAG while not degrading 2HFA-TAG. However, a non-acyl-selective lipase combined with a lesquerolyl-CoA selective DGAT could achieve the same results by regenerating any 2HFA-TAG cleaved by the lipase. The amount of labeled HFA did not decrease during the chase (Figure 4B); therefore, any HFAs removed during TAG remodeling must be reutilized for TAG biosynthesis and not further degraded. This model likely involves two separate pools of PC, one for hydroxylation and acyl editing, and a separate one for PC-derived DAG production to limit *sn*-2 HFA accumulation in TAG (Hayes and Kleiman, 1996). Precedent for multiple distinct pools of PC involved in acyl editing, de novo glycerolipid biosynthesis, and PC turnover to produce other lipids have recently been demonstrated in Arabidopsis leaves (Karki et al., 2019), and are likely part of specific metabolons of lipid metabolic enzymes spatially separated in the ER where lateral diffusion of PC acts as a “DAG carrier” between locations of de novo PC synthesis and PC turnover to produce other lipids. Likewise, recent work has indicated that AtDGAT1 and various plant DGAT2 proteins do not interact and each utilizes different pools of PC-derived DAG (but not de novo DAG) to produce TAG in Arabidopsis seeds, further suggesting distinct metabolons of plant lipid biosynthesis (Regmi et al., 2020).

### Potential enzymes of TAG remodeling through reinterpretation of *P. fendleri* transcriptomics

The isotopic tracing presented here demonstrating that TAG remodeling is the major mechanism for 2HFA-TAG accumulation in *P. fendleri* allows us to reinterpret previous developing seed transcriptomics results (Kim and Chen, 2015; Horn et al., 2016) to both clarify previous observations and suggest specific genes involved in TAG remodeling. First, the expression pattern of most lipid metabolism genes in *P. fendleri* is very similar to that of non-HFAs accumulating Brassicaceae species such as camelina that utilize a PC-derived DAG pathway of TAG synthesis (Horn et al., 2016; Yang et al., 2017), including PC:DAG cholinephosphotransferase (PDCT) a key enzyme involved in PC-derived DAG production (Kim and Chen, 2015; Horn et al., 2016). This previous result was initially confusing for two reasons: (1) to produce PC-derived DAG with *sn*-1 lesqueroleate solely by PDCT would require PC-containing lesquerolic acid as an intermediate that has not been detected (Chen et al., 2011; Supplemental Figure S1D). In addition, most plants actively keep unusual fatty acids out of membrane lipids (Millar et al., 2000); (2) high PDCT activity would be expected to produce DAG and TAG containing *sn*-2 ricinoleate (as in transgenic Arabidopsis; Hu et al., 2012) rather than the *sn*-1/3 2HFA-TAG in *P. fendleri*. However, recent work indicating multiple distinct pools of PC and DAG metabolism in the ER (Karki et al., 2019; Regmi et al., 2020) and the isotopic tracing presented here makes it clear that PDCT could produce non-HFA-containing PC-derived DAG for initial synthesis of 1HFA-TAG, which is then remodeled to 2HFA-TAG (Figure 7). Second, in castor, each Kennedy pathway enzyme selectively promotes HFA accumulation in TAG (Bafor et al., 1991; Lunn et al., 2019; Shockey et al., 2019). However, the comparison of lipid metabolic enzyme protein sequences between *P. fendleri* and eight non-HFA-accumulating Brassicaceae species suggested that the latter steps in TAG accumulation (e.g. DGAT1, DGAT2) may have evolved to specifically utilize HFA, but initial steps of the Kennedy pathway for de novo lipid assembly (e.g. GPAT9) in *P. fendleri* indicated little sequence divergence, which was unexpected if 2HFA-TAG was produced by the Kennedy pathway as in castor. The utilization of PC-derived DAG and TAG remodeling by *P. fendleri* indicates that PfeGPAT9 would not need to utilize HFA-CoA during de novo PC synthesis (Figure 7), and likely explains the lack of PfeGPAT9 coevolution with HFA accumulation.

The proposed model of TAG remodeling (Figure 7) suggests that a TAG lipase and DGAT work in tandem to remove non-HFAs from TAG and replace it with HFAs. Candidate genes involved in TAG remodeling as described below are listed in Supplemental Table S2. Castor accumulates HFA-containing TAG with the HFA selective RcDGAT2 (Burgal et al., 2008; Shockey et al., 2019), which is expressed 80-fold higher than RcDGAT1 (Troncoso-Ponce et al., 2011). However, in *P. fendleri* (and other Brassicaceae species that utilize the PC-derived DAG pathway), DGAT1 is expressed 2- to 10-fold higher than DGAT2 (Winter et al., 2007; Horn et al., 2016), thus PfeDGAT1 is likely involved in 2HFA-TAG accumulation. Previous results suggest that DGAT enzymes have the ability to acylate both *sn*-1/2 and *sn*-2/3 DAG isoforms (Wiberg et al., 1994; Eichmann et al., 2012), indicating it is plausible that PfeDGAT1 could incorporate lesquerolic acid into both positions of 2HFA-TAG through TAG remodeling. However, results indicating DGAT1s and DGAT2s have differential localization in the ER and oil body (Shockey et al., 2006; Wilfling et al., 2013), differential preferences for DAG regiochemical and molecular species substrates (Eichmann et al., 2012; Jeppson et al., 2019), and utilize different pools of DAG (Regmi et al., 2020) provide support for the intriguing possibility that PfeDGAT1 and PfeDGAT2 may be differentially involved in the *sn*-1 or *sn*-3 acylation during TAG remodeling.

Twelve genes annotated as TAG lipases are expressed in developing *P. fendleri* seeds (Horn et al., 2016). PfeTAGL-like-1 is the most highly expressed lipase during the TAG accumulation phase of embryo development and the only lipase sequence that may have coevolved with HFA accumulation (Horn et al., 2016), and the Arabidopsis homolog (Supplemental Table S2) was recently demonstrated to be enriched on oil bodies (Kretzschmar et al., 2020). Thus, PfeTAGL-like-1 is a candidate for involvement in TAG remodeling to accumulate 2HFA-TAG. The *P. fendleri* sugar-dependent 1 (*PfeSDP1*) TAG lipase is also expressed, albeit at a lower level, during seed development (Horn et al., 2016). SDP1 is the major lipase involved in TAG breakdown during oilseed germination, but SDP1 is also active during seed development leading to the reduction of oilseed TAG content during seed maturation (Eastmond, 2006; Kelly et al., 2013). Thus, PfeSDP1 could also be a candidate lipase involved in TAG remodeling. If PfeSDP1 efficiently removes HFAs from 2HFA-TAG during germination, then its role in TAG remodeling would likely be through the non-acyl-selective model discussed above. Interestingly, PfePDAT2 is highly expressed and may have coevolved with HFA accumulation (Kim and Chen, 2015; Horn et al., 2016). Considering PC does not contain lesquerolic acid, the forward PDAT reaction likely does not produce 2HFA-TAG. However, as a possible alternative to lipase-based TAG turnover in TAG remodeling, the reverse PDAT reaction (Ghosal et al., 2007) could be involved in the acyl chain removal with potential direct incorporation of oleic acid into PC for further hydroxylation. Further research on the substrate selectivity of PfeDGAT1, PfeDGAT2, PfePDAT2, PfeTAGL-like-1, PfeSDP1, and other TAG lipases; or their interaction within metabolons of lipid biosynthesis in the ER or oilbody; or the effect of their mutations on in vivo 2HFA-TAG accumulation may be needed to fully understand the enzymatic mechanisms of *P. fendleri* TAG remodeling. However, by combining isotopic tracing of lipid metabolism and gene expression data (Horn et al., 2016), we are able to propose the components of a previously unidentified TAG remodeling pathway as the major TAG biosynthetic mechanism for accumulation of membrane incompatible unusual fatty acids while utilizing DAG derived from the membrane lipid PC as a substrate for TAG synthesis.

### TAG remodeling beyond *P. fendleri*

The identification of TAG remodeling as an integral part of *P. fendleri* oil biosynthesis expands the lipid metabolic network pathways utilized by plants to accumulate a wide range of fatty acids in seed oils (Bates, 2016; Ohlrogge et al., 2018), and demonstrates that even in a developing oil storage tissue expanding TAG pools can be metabolically active through dynamic anabolic and catabolic processes. Multiple lines of evidence suggest that TAG remodeling may take place in other plants at least to varying degrees. First, in sunflower (*Helianthus annuus*), a large decrease in temperature during seed development leads to an increase in PUFA in seed oil, suggesting oleic acid in TAG may be remobilized into PC for further desaturation in an apparent inducible TAG remodeling system (Garces et al., 1994; Sarmiento et al., 1998). Second, microsomal extracts from safflower (*Carthamus tinctorius*) and castor demonstrated the enzymatic ability to exchange fatty acids between TAG molecular species (Mancha and Stymne, 1997; Stobart et al., 1997). However, in each of these plant species, the genes responsible for these observations are unknown, and the possible TAG remodeling is not considered the major pathway of TAG accumulation as it is in *P. fendleri*. Third, it is possible that TAG remodeling may not have evolved independently in *P. fendleri* for incorporation of lesquerolic acid into TAG, but that it may also occur in related Brassicaceae species, especially those that accumulate very long-chain fatty acids (e.g. ≥20 carbons) in seed oils.

As an example, we present several points of evidence about TAG biosynthesis in the Brassicaceae species *Crambe abyssinica* and rapeseed (*Brassica napus*) that is very similar to *P. fendleri*, and thus have the possibility to involve TAG remodeling. (1) TAG from both *C. abyssinica* and *B. napus* contains high levels of very long-chain fatty acids mostly erucic acid (22:1) at approximately 45%–60% of TAG and predominantly in the *sn*-1 and *sn*-3 positions, but little to no very long-chain fatty acids in PC (Gurr et al., 1972; Taylor et al., 1994; Li et al., 2012); (2) in both *C. abyssinica* and *B. napus* embryos (which actively accumulate TAG), the rapid in vivo labeling of PC (or total phospholipids) with isotopically labeled glycerol is faster than or similar to TAG labeling that is consistent with at least some of TAG biosynthesis occurring from PC-derived DAG (Gurr et al., 1974; Harris and Norton, 1988; Guan et al., 2014); (3) RNAi or mutation of PDCT in both *C. abyssinica* and *B. napus* seeds reduces the incorporation of PC-modified fatty acids (e.g. PUFA) into TAG, supporting a role for PC-derived DAG in TAG biosynthesis (Guan et al., 2015; Bai et al., 2020); (4) interestingly, RNAi of PDCT did not affect 22:1 levels in *C. abyssinica* TAG, suggesting that 22:1 is not provided by way of PDCT (Guan et al., 2015), the BnPDCT mutants were in the fatty acid elongase1 (*fae1*) mutant background and thus the effect on 22:1 was not measured (Bai et al., 2020); (5) GPAT assays from *C. abyssinica* microsomes indicated 22:1-CoA was a very poor substrate (only ∼3% as effect as 18:1-CoA), suggesting most 22:1 incorporated into the *sn*-1 position of TAG may not occur by the Kennedy pathway GPAT in *C. abyssinica* (Guan et al., 2014). The potential limited role of *C. abyssinica* GPAT for incorporation of very long-chain fatty acids into de novo DAG is similar to the *P. fendleri* result, suggesting PfeGPAT9 did not appear to evolve for HFA utilization as did later steps in TAG biosynthesis, such as PfeDGAT1 (Horn et al., 2016).

Together these previous results in both *C. abyssinica* and *B. napus* present a similar TAG biosynthetic dilemma as for *P. fendleri*, mainly how does 22:1 accumulate at the *sn*-1 position of TAG while utilizing a PC-derived DAG pathway of TAG biosynthesis, and maintaining limited amounts of 22:1 in PC? One possibility may be selective bifurcation of TAG biosynthesis between de novo DAG and PC-derived DAG with de novo-DAG-containing *sn*-1 erucic acid selectively used for de novo TAG biosynthesis rather than PC synthesis, possibly aided by a highly selective DGAT (Jeppson et al., 2019). A second possibility is that de novo DAG is rapidly transferred through PC for TAG synthesis such that little 22:1 accumulates in PC. Both potential pathways could be aided by a highly selective DGAT (Jeppson et al., 2019) that rapidly utilizes DAG containing 22:1 limiting its incorporation into other lipids. However, the poor activity of *C. abyssinica* GPAT with 22:1-CoA suggests that little DAG synthesized de novo by the Kennedy pathway would contain *sn*-1 22:1 for either direct TAG biosynthesis or PC synthesis (Guan et al., 2014). A third possibility is that the *sn*-1 erucic acid is incorporated into TAG through a TAG remodeling process as proposed here for *P. fendleri*, but has previously gone undetected in these species and possibly others.

It is highly possible that TAG remodeling could have gone undetected in *B. napus*, *C. abyssinica*, or other oil accumulating plants. First, as in Figure 2, the mass accumulation alone cannot detect changes in individual molecular species over time, that requires pulse-chase isotopic tracing. Second, the TAG remodeling measured here relied on separating and quantifying different isotopically labeled molecular species of TAG (0, 1, or 2HFA-TAG), which is relatively easily done based on the effect of polar hydroxyl groups (of HFAs) on the chromatographical separation of neutral lipids by normal phase TLC or high performance liquid chromatography (HPLC; Bafor et al., 1991; Bates and Browse, 2012; Kotapati and Bates, 2018, 2020). However, unusual fatty acids that do not affect neutral lipid polarity (e.g. erucic acid) have little effect on normal phase chromatographic separations, and thus pulse-chase radioisotopic tracing of TAG metabolism combined with normal phase separation of TAG would not have detected individual molecular species changes. The analysis of radiolabeled lipid molecular species requires more complicated reversed phase and/or argentation TLC or HPLC methods, which are more rarely combined with isotopic labeling, but can provide great insight on biosynthesis and remodeling of specific molecular species of lipids (e.g. Slack et al., 1978; Williams et al., 2000; Bates et al., 2009; Kotapati and Bates, 2020). Therefore, these previous results in both *C. abyssinica* and *B. napus* support the need for further isotopic pulse-chase tracing of TAG metabolism in other oilseed tissues with a focus on changes in TAG molecular species composition over time, and thus may reveal a broader role of TAG remodeling in determining the final oil fatty acid composition in plant seeds.

Here we demonstrate that *P. fendleri* combines the PC-derived DAG pathway for TAG synthesis with TAG remodeling as the major mechanism to accumulate membrane incompatible (but industrially valuable) unusual fatty acids in TAG, and demonstrates expanded diversity in the possible mechanisms to produce oils with diverse fatty acid composition in plants (Bates, 2016). In addition, evidence in the literature suggests that TAG remodeling may be much more wide spread, but definitive detection has remained elusive. Understanding the actual biochemical path of acyl and glycerol flux in developing oilseeds complements and allows for enhanced interpretation of transcriptomic analyses (Kim and Chen, 2015; Horn et al., 2016) to identify gene candidates involved in the control of TAG biosynthesis. The further characterization of enzymes involved in *P. fendleri* TAG remodeling will provide additional tools for the engineering of designer plant oil compositions. Including the intriguing possibility of changing seed oil composition after initial TAG synthesis, which could be key to overcoming the metabolic bottlenecks that limit unusual fatty acid flux through membrane lipids for accumulation in TAG (Bates and Browse, 2011). Future plant oil engineering strategies should consider the composition of TAG initially synthesized not as a metabolic endpoint, but as a dynamic substrate pool ripe for further manipulation.

## Materials and methods

### Plant growth and supplies

*Physaria fendleri* plants were grown at 22°C with a light intensity of ∼320 μmol m^−2^ s^−1^ and a 16-h d and 8-h night photoperiod. Flowers were hand-pollinated and tagged and the embryo development recorded as DAP. Unless otherwise indicated all chemicals were purchases from Fisher Scientific and solvents were HPLC grade or above.

### TAG and fatty acid composition analysis in developing embryos

*Physaria* *fendleri* siliques at different stages of development from 24 to 42 DAP were dissected to collect embryos. Five replicates of 10 embryos at each stage except for 24 DAP (three replicates) were used for lipid extraction and analysis. Embryos were quenched in 85°C isopropanol with 0.01% w/v butylated hydroxytoluene (BHT) for 10 min, and extracted by the hexane–isopropanol method (Hara and Radin, 1978), with modifications (Karki et al., 2019). An aliquot of total lipid was converted to fatty acid methyl esters (FAMEs) for fatty acid composition and quantification by gas chromatography with flame ionization detection (GC-FID; Karki and Bates, 2018). TAG species were separated by TLC based on the number of hydroxy fatty groups (Bates and Browse, 2011) on 20 × 20 cmSilica Gel 60 TLC plates (MilliporeSigma), stained with 0.005% w/v primulin in acetone/water (80:20, v/v) and visualized under UV. TAG bands were removed from the TLC plate and converted into FAMEs for quantification by GC-FID (Karki and Bates, 2018).

### [^14^C]acetate and [^14^C]glycerol labeling of developing embryos

*Physaria* *fendleri* embryos were collected from siliques at 30 DAP and placed into sterile growth media (Cocuron et al., 2014; Anderson, 2015; 4.41 g L^−1^ Murashige and Skoog salts, 2 mL L^−1^ Gamborg vitamin mixture, 1.4614 g L^−1^ glutamine, 14.4128 g L^−1^ glucose, 9.532 g L^−1^ HEPES, 20 μM ABA and 50% w/v PEG). The pH of the media was adjusted to 6.3 with 1M KOH. Overall, 20 μM ABA and 50% PEG w/v were added after adjusting the pH and filter sterilization. Embryos were kept in six-well plates on top of two Whatman glass microfiber filters (to prevent submersion in media) and incubated at 22°C with 30 μmol m^−2^ s^−1^ pea green light. Each isotopic labeling experiment was done at separate times with embryos isolated from different plants and involved three independent isotopic labeling replicates. Each independent time point replicates sample contained 10 embryos; thus, 30 embryos total per time point per independent isotopic labeling experiment. Embryos were preincubated for 30 min prior to isotopic labeling. To initiate the labeling, unlabeled media was removed and replaced with incubation media containing an additional 1 mM [^14^C]acetate or 0.2 mM [^14^C(U)]glycerol (American Radiochemicals Inc.). For continuous labeling, embryos were removed from media at 3, 6, 10, and 30 min and immediately quenched and lipids were extracted as described above. For pulse-chase labeling experiments, radioactive media was removed after 1 h of pulse, embryos were washed with unlabeled media five times, and incubated further in growth media without labeled substrate for the chase time points, at each time point the embryos were quenched and extracted as described above. Chase time points of 0, 2, 24, 74, 120, and 168 h and 0, 2, 24, 72, and 120 h for [^14^C]acetate and [^14^C]glycerol pulse chase, respectively.

### Analysis of radiolabeled lipids

Total radioactivity in lipid extracts was measured by liquid scintillation counting and total lipid content by GC-FID of FAME. For [^14^C]glycerol-labeled samples, the lipid glycerol backbone was separated from the acyl groups by conversion to FAME and phase partitioning into aqueous and hexane phases, with liquid scintillation counting of the aqueous phase as the glycerol backbones and the hexane phase as the acyl groups (Bates and Browse, 2011). PLs were separated by TLC in chloroform/methanol/acetic acid (75:25:8, v/v/v). Neutral lipids were separated as described above (TLC example; Supplemental Figure S7). All plates were stained with primulin as described above to visualize mass bands and mark nonradioactive standards. [^14^C]acetate-labeled lipids were quantified by phosphor imaging of the TLC plate on a GE Typhoon FLA 7000 phosphor imager and radioactivity was quantified by ImageQuant software version 7.0. All [^14^C]glycerol-labeled lipids were removed from the TLC plate, converted to FAMEs, and liquid scintillation was counted as described above.

### Analysis of radiolabeled acyl groups

To determine the relative accumulation of [^14^C]acetate-labeled HFAs and non-HFAs, total lipid FAMEs were separated by TLC developed in hexanes/diethyl ether/acetic acid (70:30:1, v/v/v) and quantified by phosphor imaging as described above. For fatty acid compositions of individual lipids, the lipids separated by TLC (as above) were converted to FAMEs and subsequently separated by Argentation TLC (Pollard et al., 2015), stained with primulin and radioactivity quantified by phosphor imaging. PC stereochemistry and analysis of labeled products by phosphor imaging were done as previously reported (Bates et al., 2007).

### Statistical analysis

All graphing, linear regression, curve fitting, and statistical analysis were done in GraphPad Prism version 9.

### Accession numbers

Accession numbers to the referenced genes are in Supplemental Table S2.

## Supplemental data

The following materials are available in the online version of this article. 

**Supplemental Figure S1**. Fatty acid composition of different lipid molecular species across *P. fendleri* embryo development.

**Supplemental Figure S2**. Total lipid ^14^C accumulation from continuous [^14^C]acetate feeding of *P. fendleri* embryos.

**Supplemental Figure S3.** Total fatty acid mass accumulation during [^14^C]acetate pulse-chase labeling of developing *P. fendleri* embryos.

**Supplemental Figure S4**. Total fatty acid mass accumulation during [^14^C]glycerol pulse-chase labeling of developing *P. fendleri* embryos.

**Supplemental Figure S5**. Rates of individual lipid mass accumulation in developing *P. fendleri* embryos.

**Supplemental Figure S6**. Fitting of [^14^C]glycerol pulse-chase labeling data (Figure 6D) to one phase exponential decay curves.

**Supplemental Figure S7**. Representative phosphor image for total lipid separation.

**Supplemental Table S1**. Increase in polyunsaturated fatty acids and 20:1 during [^14^C]acetate chase 24–168 h as compared to substrates available in PC.

**Supplemental Table S2**. Potential *P. fendleri* genes involved in TAG remodeling.

## Supplementary Material

kiab294_Supplementary_Data

## Notes

P.D.B. conceived the project and procured the funding; S.B. and P.D.B. designed the experiments; S.B. performed the experiments; S.B and P.D.B. analyzed the data; S.B. and P.D.B. wrote the article; P.D.B. agrees to serve as the author responsible for contact and ensures communication.

The author responsible for distribution of materials integral to the findings presented in this article in accordance with the policy described in the Instructions for Authors (https://academic.oup.com/plphys/pages/general-instructions) is: Philip D. Bates (phil_bates@wsu.edu).

## References

[kiab294-B1] Al-Shehbaz IA , O'Kane SL Jr (2002) Lesquerella is united with Physaria (Brassicaceae). Novon 12**:** 319–329

[kiab294-B2] Allen DK , Bates PD, Tjellström H (2015) Tracking the metabolic pulse of plant lipid production with isotopic labeling and flux analyses: past, present and future. Prog Lipid Res 58**:** 97–120 25773881 10.1016/j.plipres.2015.02.002

[kiab294-B3] Anderson B (2015) 13C Labeling of the Tricarboxylic Acid Cycle and Carbon Conversion Efficiency in Lesquerella (Physaria fendleri) Embryos. The Ohio State University, Ohio

[kiab294-B4] Aryal N , Lu C (2018) A phospholipase C-like protein from Ricinus communis increases hydroxy fatty acids accumulation in transgenic seeds of Camelina sativa. Front Plant Sci 9**:** 1576–1576 30443260 10.3389/fpls.2018.01576 PMC6221933

[kiab294-B5] Bafor M , Smith MA, Jonsson L, Stobart K, Stymne S (1991) Ricinoleic acid biosynthesis and triacylglycerol assembly in microsomal preparations from developing castor-bean (Ricinus-communis) endosperm. Biochem J 280**:** 507–514 1747126 10.1042/bj2800507 PMC1130577

[kiab294-B6] Bai S , Wallis JG, Denolf P, Engelen S, Bengtsson JD, Van Thournout M, Dierickx J, Haesendonckx B, Browse J (2020) The biochemistry of headgroup exchange during triacylglycerol synthesis in canola. Plant J 103**:** 83–94 31991038 10.1111/tpj.14709 PMC7605783

[kiab294-B7] Bao XM , Pollard M, Ohlrogge J (1998) The biosynthesis of erucic acid in developing embryos of Brassica rapa. Plant Physiol 118**:** 183–190 9733537 10.1104/pp.118.1.183 PMC34854

[kiab294-B8] Bates PD (2016) Understanding the control of acyl flux through the lipid metabolic network of plant oil biosynthesis. Biochim Biophys Acta 1861**:** 1214–1225 27003249 10.1016/j.bbalip.2016.03.021

[kiab294-B9] Bates PD , Browse J (2011) The pathway of triacylglycerol synthesis through phosphatidylcholine in Arabidopsis produces a bottleneck for the accumulation of unusual fatty acids in transgenic seeds. Plant J 68**:** 387–399 21711402 10.1111/j.1365-313X.2011.04693.x

[kiab294-B10] Bates PD , Browse J (2012) The significance of different diacylgycerol synthesis pathways on plant oil composition and bioengineering. Front Plant Sci 3**:** 147 22783267 10.3389/fpls.2012.00147 PMC3387579

[kiab294-B11] Bates PD , Durrett TP, Ohlrogge JB, Pollard M (2009) Analysis of acyl fluxes through multiple pathways of triacylglycerol synthesis in developing soybean embryos. Plant Physiol 150**:** 55–72 19329563 10.1104/pp.109.137737 PMC2675710

[kiab294-B12] Bates PD , Fatihi A, Snapp AR, Carlsson AS, Browse J, Lu C (2012) Acyl editing and headgroup exchange are the major mechanisms that direct polyunsaturated fatty acid flux into triacylglycerols. Plant Physiol 160**:** 1530–1539 22932756 10.1104/pp.112.204438 PMC3490606

[kiab294-B13] Bates PD , Johnson SR, Cao X, Li J, Nam J-W, Jaworski JG, Ohlrogge JB, Browse J (2014) Fatty acid synthesis is inhibited by inefficient utilization of unusual fatty acids for glycerolipid assembly. Proc Natl Acad Sci USA 111**:** 1204–1209 24398521 10.1073/pnas.1318511111 PMC3903203

[kiab294-B14] Bates PD , Ohlrogge JB, Pollard M (2007) Incorporation of newly synthesized fatty acids into cytosolic glycerolipids in pea leaves occurs via acyl editing. J Biol Chem 282**:** 31206–31216 17728247 10.1074/jbc.M705447200

[kiab294-B15] Bradberry SM , Dickers KJ, Rice P, Griffiths GD, Vale JA (2003) Ricin poisoning. Toxicol Rev 22**:** 65–70 14579548 10.2165/00139709-200322010-00007

[kiab294-B16] Broun P , Boddupalli S, Somerville C (1998) A bifunctional oleate 12-hydroxylase: desaturase from Lesquerella fendleri. Plant J 13**:** 201–210 9680976 10.1046/j.1365-313x.1998.00023.x

[kiab294-B17] Broun P , Shanklin J, Whittle E, Somerville C (1998) Catalytic plasticity of fatty acid modification enzymes underlying chemical diversity of plant lipids. Science 282**:** 1315–1317 9812895 10.1126/science.282.5392.1315

[kiab294-B18] Broun P , Somerville C (1997) Accumulation of ricinoleic, lesquerolic, and densipolic acids in seeds of transgenic Arabidopsis plants that express a fatty acyl hydroxylase cDNA from castor bean. Plant Physiol 113**:** 933–942 9085577 10.1104/pp.113.3.933 PMC158213

[kiab294-B19] Burgal J , Shockey J, Lu C, Dyer J, Larson T, Graham I, Browse J (2008) Metabolic engineering of hydroxy fatty acid production in plants: RcDGAT2 drives dramatic increases in ricinoleate levels in seed oil. Plant Biotechnol J 6**:** 819–831 18643899 10.1111/j.1467-7652.2008.00361.x PMC2908398

[kiab294-B20] Carlsson AS , Yilmaz JL, Green AG, Stymne S, Hofvander P (2011) Replacing fossil oil with fresh oil - with what and for what? Eur J Lipid Sci Technol 113**:** 812–831 22102794 10.1002/ejlt.201100032 PMC3210827

[kiab294-B21] Chen G , Woodfield H, Pan X, Harwood J, Weselake R (2015) Acyl-trafficking during plant oil accumulation. Lipids 50**:** 1057–1068 26459450 10.1007/s11745-015-4069-x

[kiab294-B22] Chen GQ , Lin J-T, Lu C (2011) Hydroxy fatty acid synthesis and lipid gene expression during seed development in Lesquerella fendleri. Ind Crops Prod 34**:** 1286–1292

[kiab294-B23] Chen GQ , Lin JT, van Erp H, Johnson K, Lu CF (2020) Regiobiochemical analysis reveals the role of castor LPAT2 in the accumulation of hydroxy fatty acids in transgenic lesquerella seeds. Biocatal Agric Biotechnol 25**:** 101617

[kiab294-B24] Chen GQ , Vang L, Lin J-T (2009) Seed development in Lesquerella fendleri (L.). HortScience 44**:** 1415–1418

[kiab294-B25] Cocuron J-C , Anderson B, Boyd A, Alonso AP (2014) Targeted metabolomics of Physaria fendleri, an industrial crop producing hydroxy fatty acids. Plant Cell Physiol 55**:** 620–633 24443498 10.1093/pcp/pcu011

[kiab294-B26] Correa SM , Fernie AR, Nikoloski Z, Brotman Y (2020) Towards model-driven characterization and manipulation of plant lipid metabolism. Prog Lipid Res 80**:** 101051 32640289 10.1016/j.plipres.2020.101051

[kiab294-B27] Dauk M , Lam P, Kunst L, Smith MA (2007) A FAD2 homologue from Lesquerella lindheimeri has predominantly fatty acid hydroxylase activity. Plant Sci 173**:** 43–49

[kiab294-B28] Dauk M , Lam P, Smith MA (2009) The role of diacylglycerol acyltransferase-1 and phospholipid:diacylglycerol acyltransferase-1 and-2 in the incorporation of hydroxy fatty acids into triacylglycerol in Arabidopsis thaliana expressing a castor bean oleate 12-hydroxylase gene. Botany-Botanique 87**:** 552–560

[kiab294-B29] Dyer JM , Stymne S, Green AG, Carlsson AS (2008) High-value oils from plants. The Plant J 54**:** 640–655 18476869 10.1111/j.1365-313X.2008.03430.x

[kiab294-B30] Eastmond PJ (2006) SUGAR-DEPENDENT1 Encodes a patatin domain triacylglycerol lipase that initiates storage oil breakdown in germinating Arabidopsis seeds. Plant Cell 18: 665–675 16473965 10.1105/tpc.105.040543 PMC1383641

[kiab294-B31] Eichmann TO , Kumari M, Haas JT, Farese RV, Zimmermann R, Lass A, Zechner R (2012) Studies on the substrate and stereo/regioselectivity of adipose triglyceride lipase, hormone-sensitive lipase, and diacylglycerol-O-acyltransferases. J Biol Chem 287**:** 41446–41457 23066022 10.1074/jbc.M112.400416 PMC3510842

[kiab294-B32] Gao Q , Goodman J (2015) The lipid droplet—a well-connected organelle. Front Cell Dev Biol 3: 49 26322308 10.3389/fcell.2015.00049 PMC4533013

[kiab294-B33] Garces R , Sarmiento C, Mancha M (1994) Oleate from triacylglycerols is desaturated in cold-induced developing sunflower (Helianthus annuus L.) seeds. Planta 193**:** 473–477

[kiab294-B34] Ghosal A , Banas A, Stahl U, Dahlqvist A, Lindqvist Y, Stymne S (2007) Saccharomyces cerevisiae phospholipid: diacylglycerol acyl transferase (PDAT) devoid of its membrane anchor region is a soluble and active enzyme retaining its substrate specificities. Biochim Biophys Acta 1771**:** 1457–1463 18037386 10.1016/j.bbalip.2007.10.007

[kiab294-B35] Guan R , Lager I, Li X, Stymne S, Zhu L-H (2014) Bottlenecks in erucic acid accumulation in genetically engineered ultrahigh erucic acid Crambe abyssinica. Plant Biotechnol J 12**:** 193–203 24119222 10.1111/pbi.12128 PMC4286110

[kiab294-B36] Guan R , Li X, Hofvander P, Zhou XR, Wang D, Stymne S, Zhu LH (2015) RNAi targeting putative genes in phosphatidylcholine turnover results in significant change in fatty acid composition in Crambe abyssinica seed oil. Lipids 50**:** 407–416 25753896 10.1007/s11745-015-4004-1

[kiab294-B37] Gurr MI , Blades J, Appleby RS (1972) Studies on seed-oil triglycerides. Eur J Biochem 29**:** 362–368 5081618 10.1111/j.1432-1033.1972.tb01997.x

[kiab294-B38] Gurr MI , Blades J, Appleby RS, Smith CG, Robinson MP, Nichols BW (1974) Studies on seed‐oil triglycerides: triglyceride biosynthesis and storage in whole seeds and oil bodies of Crambe abyssinica. Eur J Biochem 43**:** 281–290 4365180 10.1111/j.1432-1033.1974.tb03411.x

[kiab294-B39] Hara A , Radin NS (1978) Lipid extraction of tissues with a low-toxicity solvent. Anal Biochem 90**:** 420–426 727482 10.1016/0003-2697(78)90046-5

[kiab294-B40] Harris JF , Norton C (1988) Triacylglycerol biosynthesis in high erucate rapeseed. *In* 7th International Rapeseed Congress/Convened under the Patronage of Stanislaw Zieba; by the Plant Breeding and Acclimatization Institute under the Auspices of the Group Consultatif International de Recherche sur le Colza. Panstwowe Wydawnictwo Rolnicze i Lesne, Poznan.

[kiab294-B41] Hayes D , Kleiman R, Phillips B (1995) The triglyceride composition, structure, and presence of estolides in the oils of*Lesquerella* and related species. J Am Oil Chem' Soc 72**:** 559–569

[kiab294-B42] Hayes DG , Kleiman R (1996) A detailed triglyceride analysis ofLesquerella fendleri oil: column chromatographic fractionation followed by supercritical fluid chromatography. J Am Oil Chem' Soc 73**:** 267–269

[kiab294-B43] Horn PJ , Liu J, Cocuron JC, McGlew K, Thrower NA, Larson M, Lu C, Alonso AP, Ohlrogge J (2016) Identification of multiple lipid genes with modifications in expression and sequence associated with the evolution of hydroxy fatty acid accumulation in Physaria fendleri. Plant J 86**:** 322–348 26991237 10.1111/tpj.13163

[kiab294-B44] Hu Z , Ren Z, Lu C (2012) The phosphatidylcholine diacylglycerol cholinephosphotransferase is required for efficient hydroxy fatty acid accumulation in transgenic Arabidopsis. Plant Physiol 158**:** 1944–1954 22371508 10.1104/pp.111.192153 PMC3320197

[kiab294-B45] Jeppson S , Demski K, Carlsson AS, Zhu L-H, Banaś A, Stymne S, Lager I (2019) Crambe hispanica Subsp. abyssinica diacylglycerol acyltransferase specificities towards diacylglycerols and acyl-CoA reveal combinatorial effects that greatly affect enzymatic activity and specificity. Front Plant Sci 10**:** 1442 31798607 10.3389/fpls.2019.01442 PMC6863138

[kiab294-B46] Karki N , Bates PD (2018) The effect of light conditions on interpreting oil composition engineering in Arabidopsis seeds. Plant Direct 2**:** e00067 31245729 10.1002/pld3.67 PMC6508571

[kiab294-B47] Karki N , Johnson BS, Bates PD (2019) Metabolically distinct pools of phosphatidylcholine are involved in trafficking of fatty acids out of and into the chloroplast for membrane production. Plant Cell 31**:** 2768–2788 31511316 10.1105/tpc.19.00121 PMC6881139

[kiab294-B48] Kelly AA , Shaw E, Powers SJ, Kurup S, Eastmond PJ (2013) Suppression of the SUGAR-DEPENDENT1 triacylglycerol lipase family during seed development enhances oil yield in oilseed rape (Brassica napus L.). Plant Biotechnol J 11**:** 355–361 23171303 10.1111/pbi.12021

[kiab294-B49] Kim HU , Chen GQ (2015) Identification of hydroxy fatty acid and triacylglycerol metabolism-related genes in lesquerella through seed transcriptome analysis. BMC Genomics 16**:** 230 25881190 10.1186/s12864-015-1413-8 PMC4381405

[kiab294-B50] Kotapati HK , Bates PD (2018) A normal phase high performance liquid chromatography method for the separation of hydroxy and non-hydroxy neutral lipid classes compatible with ultraviolet and in-line liquid scintillation detection of radioisotopes. J Chromatogr B 1102–1103**:** 52–59 10.1016/j.jchromb.2018.10.012 30368043

[kiab294-B51] Kotapati HK , Bates PD (2020) Analysis of isotopically-labeled monogalactosyldiacylglycerol molecular species from [14C]acetate-labeled tobacco leaves. Bio Protoc 10**:** e3864 10.21769/BioProtoc.3864 PMC7842291 33659505

[kiab294-B52] Kotapati HK , Bates PD (2020) Normal phase HPLC method for combined separation of both polar and neutral lipid classes with application to lipid metabolic flux. J Chromatogr B 1145**:** 122099 10.1016/j.jchromb.2020.122099 32305707

[kiab294-B53] Kretzschmar FK , Doner NM, Krawczyk HE, Scholz P, Schmitt K, Valerius O, Braus GH, Mullen RT, Ischebeck T (2020) Identification of low-abundance lipid droplet proteins in seeds and seedlings. Plant Physiol 182**:** 1326–1345 31826923 10.1104/pp.19.01255 PMC7054876

[kiab294-B54] Lager I , Yilmaz JL, Zhou X-R, Jasieniecka K, Kazachkov M, Wang P, Zou J, Weselake R, Smith MA, Bayon S, et al (2013) Plant acyl-CoA:Lysophosphatidylcholine Acyltransferases (LPCATs) have different specificities in their forward and reverse reactions. J Biol Chem 288**:** 36902–36914 24189065 10.1074/jbc.M113.521815 PMC3873549

[kiab294-B55] Lee K-R , Chen G, Kim H (2015) Current progress towards the metabolic engineering of plant seed oil for hydroxy fatty acids production. Plant Cell Rep 34: 603–615 25577331 10.1007/s00299-015-1736-6

[kiab294-B56] Li-Beisson Y , Shorrosh B, Beisson F, Andersson MX, Arondel V, Bates PD, Baud S, Bird D, Debono A, Durrett TP, et al (2013) Acyl-lipid metabolism. Arabidopsis Book 11**:** e0161 23505340 10.1199/tab.0161 PMC3563272

[kiab294-B57] Li X , van Loo EN, Gruber J, Fan J, Guan R, Frentzen M, Stymne S, Zhu LH (2012) Development of ultra-high erucic acid oil in the industrial oil crop Crambe abyssinica. Plant Biotechnol J 10**:** 862–870 22642539 10.1111/j.1467-7652.2012.00709.x

[kiab294-B58] Lin J-T , Chen GQ (2013) Identification of TAG and DAG and their FA constituents in lesquerella (Physaria fendleri) oil by HPLC and MS. J Am Oil Chem' Soc 90**:** 1819–1829

[kiab294-B59] Lin J-T , Chen GQ (2014) Quantification of the molecular species of TAG and DAG in Lesquerella (Physaria fendleri) oil by HPLC and MS. J Am Oil Chem' Soc 91**:** 1417–1424

[kiab294-B60] Lin J-T , Fagerquist CK, Chen GQ (2015) Ratios of Regioisomers of the molecular species of triacylglycerols in Lesquerella (Physaria fendleri) oil estimated by mass spectrometry. J Am Oil Chem' Soc 93: 1–9

[kiab294-B61] Lu C , Xin Z, Ren Z, Miquel M, Browse J (2009) An enzyme regulating triacylglycerol composition is encoded by the ROD1 gene of Arabidopsis. Proc Natl Acad Sci USA 106**:** 18837–18842 19833868 10.1073/pnas.0908848106 PMC2774007

[kiab294-B62] Lu CF , Fulda M, Wallis JG, Browse J (2006) A high-throughput screen for genes from castor that boost hydroxy fatty acid accumulation in seed oils of transgenic Arabidopsis. Plant J 45**:** 847–856 16460516 10.1111/j.1365-313X.2005.02636.x

[kiab294-B63] Lunn D , Wallis JG, Browse J (2019) Tri-hydroxy-triacylglycerol is efficiently produced by position-specific castor acyltransferases. Plant Physiol 179**:** 1050–1063 30610110 10.1104/pp.18.01409 PMC6393782

[kiab294-B64] Mancha M , Stymne S (1997) Remodelling of triacylglycerols in microsomal preparations from developing castor bean (Ricinus communis L) endosperm. Planta 203**:** 51–57

[kiab294-B65] Millar AA , Smith MA, Kunst L (2000) All fatty acids are not equal: discrimination in plant membrane lipids. Trends Plant Sci 5**:** 95–101 10707074 10.1016/s1360-1385(00)01566-1

[kiab294-B66] Moon H , Smith MA, Kunst L (2001) A condensing enzyme from the seeds of Lesquerella fendleri that specifically elongates hydroxy fatty acids. Plant Physiol 127**:** 1635–1643 11743108 PMC133568

[kiab294-B67] Mutlu H , Meier MAR (2010) Castor oil as a renewable resource for the chemical industry. Eur J Lipid Sci Technol 112**:** 10–30

[kiab294-B68] Napier JA (2007) The production of unusual fatty acids in transgenic plants. Annu Rev Plant Biol 58**:** 295–319 17472567 10.1146/annurev.arplant.58.032806.103811

[kiab294-B69] Nguyen HT , Park H, Koster KL, Cahoon RE, Nguyen HTM, Shanklin J, Clemente TE, Cahoon EB (2015) Redirection of metabolic flux for high levels of omega-7 monounsaturated fatty acid accumulation in camelina seeds. Plant Biotechnol J 13**:** 38–50 25065607 10.1111/pbi.12233

[kiab294-B70] Ohlrogge J , Browse J (1995) Lipid biosynthesis. Plant Cell 7**:** 957–970 7640528 10.1105/tpc.7.7.957 PMC160893

[kiab294-B71] Ohlrogge J , Thrower N, Mhaske V, Stymne S, Baxter M, Yang W, Liu J, Shaw K, Shorrosh B, Zhang M, et al (2018) PlantFAdb: a resource for exploring hundreds of plant fatty acid structures synthesized by thousands of plants and their phylogenetic relationships. Plant J 96**:** 1299–1308 30242919 10.1111/tpj.14102

[kiab294-B72] Pollard M , Delamarter D, Martin TM, Shachar-Hill Y (2015) Lipid labeling from acetate or glycerol in cultured embryos of Camelina sativa seeds: a tale of two substrates. Phytochemistry 118**:** 192–203 26265565 10.1016/j.phytochem.2015.07.021

[kiab294-B73] Pollard M , Martin TM, Shachar-Hill Y (2015) Lipid analysis of developing Camelina sativa seeds and cultured embryos. Phytochemistry 118**:** 23–32 26262674 10.1016/j.phytochem.2015.07.022

[kiab294-B74] Pollard MR , Stumpf PK (1980) Long-chain (C-20 and C-22) fatty-acid biosynthesis in developing seeds of Tropaeolum-majus - an in vivo study. Plant Physiol 66**:** 641–648 16661495 10.1104/pp.66.4.641 PMC440696

[kiab294-B75] Pyc M , Cai Y, Greer MS, Yurchenko O, Chapman KD, Dyer JM, Mullen RT (2017) Turning over a new leaf in lipid droplet biology. Trends Plant Sci 22**:** 596–609 28454678 10.1016/j.tplants.2017.03.012

[kiab294-B76] Reed DW , Taylor DC, Covello PS (1997) Metabolism of hydroxy fatty acids in developing seeds in the genera Lesquerella (Brassicaceae) and Linum (Linaceae). Plant Physiol 114**:** 63–68 12223689 10.1104/pp.114.1.63 PMC158279

[kiab294-B77] Regmi A , Shockey J, Kotapati HK, Bates PD (2020) Oil-producing metabolons containing DGAT1 use separate substrate pools from those containing DGAT2 or PDAT. Plant Physiol 184**:** 720–737 32732347 10.1104/pp.20.00461 PMC7536707

[kiab294-B78] Roughan PG , Slack CR (1982) Cellular-organization of glycerolipid metabolism. Annu Rev Plant Physiol Plant Mol Biol 33**:** 97–132

[kiab294-B79] Sarmiento C , Garces R, Mancha M (1998) Oleate desaturation and acyl turnover in sunflower (Helianthus annuus L.) seed lipids during rapid temperature adaptation. Planta 205**:** 595–600

[kiab294-B80] Segel IH (1976) Biochemical Calculations, Ed 2. John Wiley & Sons, New York

[kiab294-B81] Shanklin J , Cahoon EB (1998) Desaturation and related modifications of fatty acids. Annu Rev Plant Physiol Plant Mol Biol 49**:** 611–641 15012248 10.1146/annurev.arplant.49.1.611

[kiab294-B82] Shockey J , Lager I, Stymne S, Kotapati HK, Sheffield J, Mason C, Bates PD (2019) Specialized lysophosphatidic acid acyltransferases contribute to unusual fatty acid accumulation in exotic Euphorbiaceae seed oils. Planta 249**:** 1285–1299 30610363 10.1007/s00425-018-03086-y

[kiab294-B83] Shockey J , Regmi A, Cotton K, Adhikari N, Browse J, Bates PD (2016) Identification of Arabidopsis GPAT9 (At5g60620) as an essential gene involved in triacylglycerol biosynthesis. Plant Physiol 170**:** 163–179 26586834 10.1104/pp.15.01563 PMC4704598

[kiab294-B84] Shockey JM , Gidda SK, Chapital DC, Kuan J-C, Dhanoa PK, Bland JM, Rothstein SJ, Mullen RT, Dyer JM (2006) Tung tree DGAT1 and DGAT2 have nonredundant functions in triacylglycerol biosynthesis and are localized to different subdomains of the endoplasmic reticulum. Plant Cell Online 18**:** 2294–2313 10.1105/tpc.106.043695 PMC1560902 16920778

[kiab294-B85] Sin C , Chiarugi D, Valleriani A (2016) Degradation parameters from pulse-chase experiments. PLoS One 11**:** e0155028 27182698 10.1371/journal.pone.0155028 PMC4868333

[kiab294-B86] Slack CR , Roughan PG, Balasingham N (1978) Labeling of glycerolipids in cotyledons of developing oilseeds by [1-C-14]acetate and [2-H-3]glycerol. Biochem J 170**:** 421–433 580379 10.1042/bj1700421 PMC1183910

[kiab294-B87] Smith MA , Moon H, Chowrira G, Kunst L (2003) Heterologous expression of a fatty acid hydroxylase gene in developing seeds of Arabidopsis thaliana. Planta 217**:** 507–516 14520576 10.1007/s00425-003-1015-6

[kiab294-B88] Stobart K , Mancha M, Lenman M, Dahlqvist A, Stymne S (1997) Triacylglycerols are synthesised and utilized by transacylation reactions in microsomal preparations of developing safflower (Carthamus tinctorius L) seeds. Planta 203**:** 58–66

[kiab294-B89] Taylor DC , MacKenzie SL, McCurdy AR, McVetty PBE, Giblin EM, Pass EW, Stone SJ, Scarth R, Rimmer SR, Pickard MD (1994) Stereospecific analyses of seed triacylglycerols from high-erucic acid brassicaceae: Detection of erucic acid at thesn-2 position inBrassica oleracea L. Genotypes. J Am Oil Chem' Soc 71**:** 163–167

[kiab294-B90] Troncoso-Ponce MA , Kilaru A, Cao X, Durrett TP, Fan J, Jensen JK, Thrower NA, Pauly M, Wilkerson C, Ohlrogge JB (2011) Comparative deep transcriptional profiling of four developing oilseeds. Plant J 68**:** 1014–1027 21851431 10.1111/j.1365-313X.2011.04751.x PMC3507003

[kiab294-B91] van Erp H , Bates PD, Burgal J, Shockey J, Browse J (2011) Castor phospholipid: diacylglycerol acyltransferase facilitates efficient metabolism of hydroxy fatty acids in transgenic Arabidopsis. Plant Physiol 155**:** 683–693 21173026 10.1104/pp.110.167239 PMC3032459

[kiab294-B92] van Erp H , Shockey J, Zhang M, Adhikari ND, Browse J (2015) Reducing isozyme competition increases target fatty acid accumulation in seed triacylglycerols of transgenic Arabidopsis. Plant Physiol 168**:** 36–46 25739701 10.1104/pp.114.254110 PMC4424008

[kiab294-B93] Vandeloo FJ , Broun P, Turner S, Somerville C (1995) An oleate 12-hydroxylase from *Ricinus communis* L. is a fatty acyl desaturase homolog. Proc Natl Acad Sci USA 92**:** 6743–6747 7624314 10.1073/pnas.92.15.6743 PMC41405

[kiab294-B94] Wiberg E , Tillberg E, Stymne S (1994) Substrates of diacylglycerol acyltransferase in microsomes from developing oil seeds. Phytochemistry 36**:** 573–577

[kiab294-B95] Wilfling F , Wang H, Haas JT, Krahmer N, Gould TJ, Uchida A, Cheng JX, Graham M, Christiano R, Frohlich F, et al (2013) Triacylglycerol synthesis enzymes mediate lipid droplet growth by relocalizing from the ER to lipid droplets. Dev Cell 24**:** 384–399 23415954 10.1016/j.devcel.2013.01.013 PMC3727400

[kiab294-B96] Williams JP , Imperial V, Khan MU, Hodson JN (2000) The role of phosphatidylcholine in fatty acid exchange and desaturation in Brassica napus L. leaves. Biochem J 349**:** 127–133 10861220 10.1042/0264-6021:3490127 PMC1221129

[kiab294-B97] Winter D , Vinegar B, Nahal H, Ammar R, Wilson GV, Provart NJ (2007) An “Electronic Fluorescent Pictograph” browser for exploring and analyzing large-scale biological data sets. PLoS One 2**:** e718 17684564 10.1371/journal.pone.0000718 PMC1934936

[kiab294-B98] Yang W , Wang G, Li J, Bates PD, Wang X, Allen DK (2017) Phospholipase Dzeta enhances diacylglycerol flux into triacylglycerol. Plant Physiol 174**:** 110–123 28325849 10.1104/pp.17.00026 PMC5411150

[kiab294-B99] Yoon K , Han D, Li Y, Sommerfeld M, Hu Q (2012) Phospholipid:diacylglycerol acyltransferase is a multifunctional enzyme involved in membrane lipid turnover and degradation while synthesizing triacylglycerol in the unicellular green microalga Chlamydomonas reinhardtii. Plant Cell 24**:** 3708–3724 23012436 10.1105/tpc.112.100701 PMC3480297

[kiab294-B100] Yu XH , Cahoon RE, Horn PJ, Shi H, Prakash RR, Cai Y, Hearney M, Chapman KD, Cahoon EB, Schwender J, et al (2018) Identification of bottlenecks in the accumulation of cyclic fatty acids in camelina seed oil. Plant Biotechnol J 16**:** 926–938 28929610 10.1111/pbi.12839 PMC5866947

