# Supplementary material for: Triacylglycerol remodeling in Physaria fendleri indicates oil accumulation is dynamic and not a metabolic endpoint
Source: Plant Physiol. 2021 Jun 24;187(2):799–815. doi: 10.1093/plphys/kiab294 (PMC8491037; doi:10.1093/plphys/kiab294)
Supplement: kiab294_Supplementary_Data [file kiab294_supplementary_data.pdf]

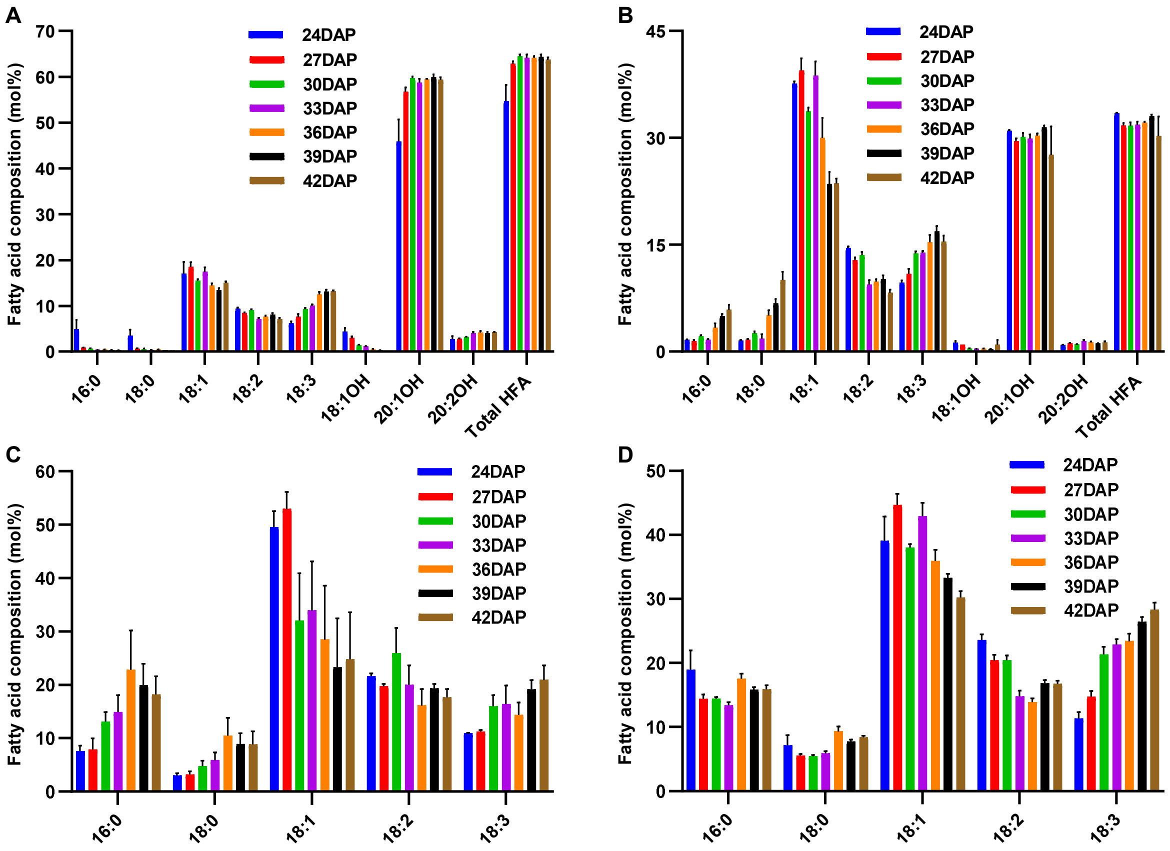

Supplemental Figure S1. Fatty acid composition of different lipid molecular species across *P. fendleri* embryo development. A) 2HFA-TAG B) 1HFA-TAG C) 0HFA-TAG and D) PC. Data is mean ± SEM of 4 replicates for each stage except for 24DAP, 3 replicates.

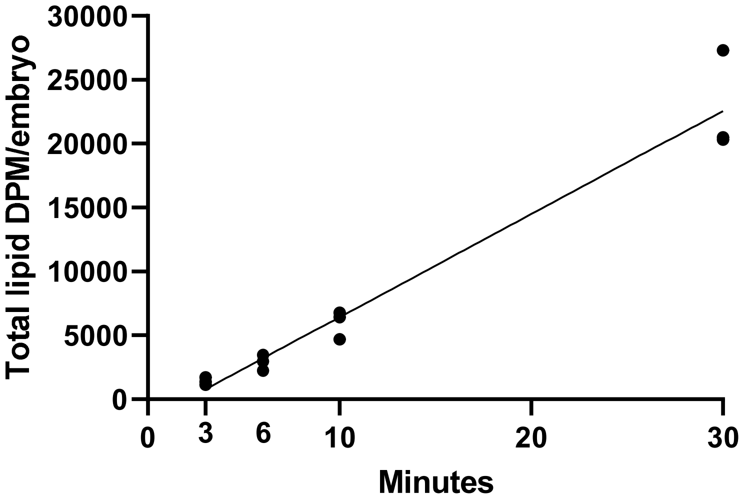

Supplemental Figure S2. Total lipid ^{14}C accumulation from continuous [^{14}C]acetate feeding of *P. fendleri* embryos. Three individual labeling replicates of 10 embryos per replicate at each time point.,120 total embryos. Line is the linear regression.

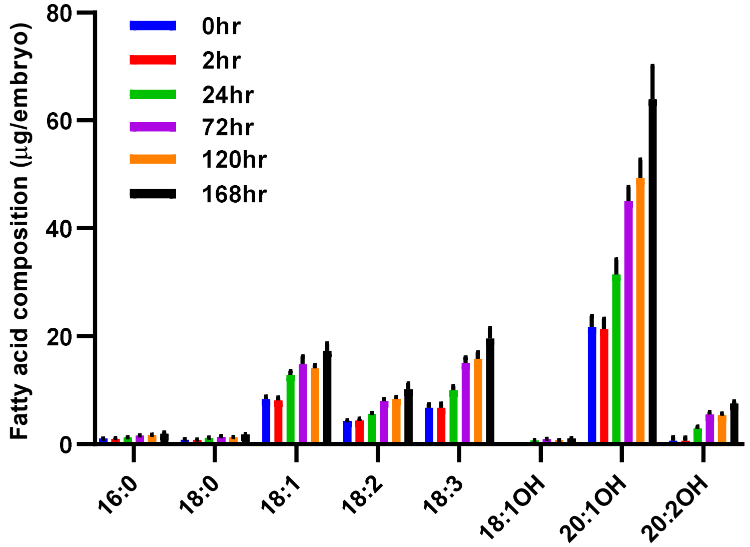

Supplemental Figure S3. Total fatty acid mass accumulation during [^{14}C]acetate pulse-chase labeling of developing *P. fendleri* embryos. Data is mean ± SEM of 3 individual labeling replicates of ten embryos for each time point replicate, 180 total embryos for the time course.

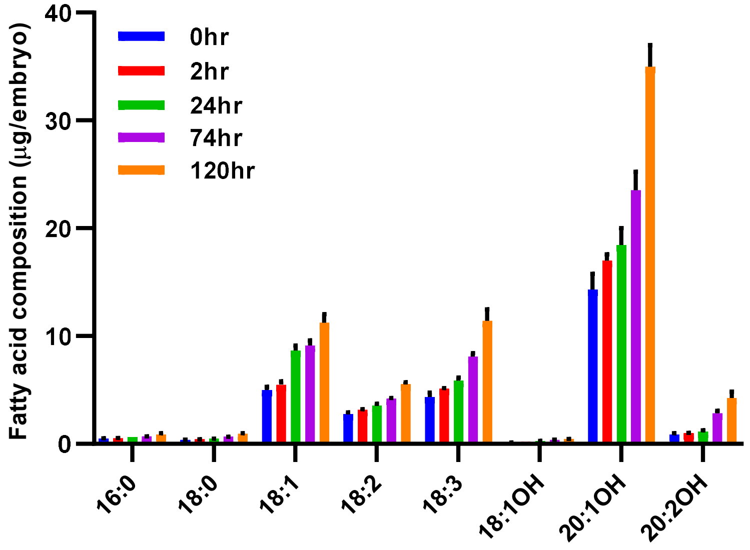

Supplemental Figure S4. Total fatty acid mass accumulation during [14C]glycerol pulse-chase labeling of developing *P. fendleri* embryos. Data is mean ± SEM of 3 individual labeling replicates of ten embryos for each time point replicate, 150 total embryos for the time course.

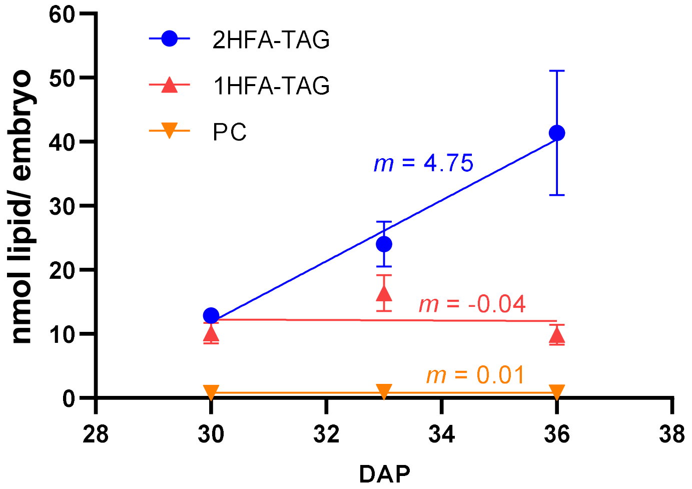

Supplemental Figure S5. Rates of individual lipid mass accumulation in developing *P. fendleri* embryos. The lipid accumulation in main text Figure 2 and Figure S1 was converted to nmol individual lipid based on weight to mole conversion factors for FAME (Christie WW), and adjusting for three fatty acids in each TAG and two in PC. The rate of accumulation is determined from the slope of each linear regression. Data is mean ± SEM of 4 individual replicates.

Christie WW (2003) Lipid Analysis: Isolation, Separation, Identification and Structural Analysis of Lipids, Ed 3rd. The Oily Press an imprint of PJ Barnes & Associates, Bridgwater, England

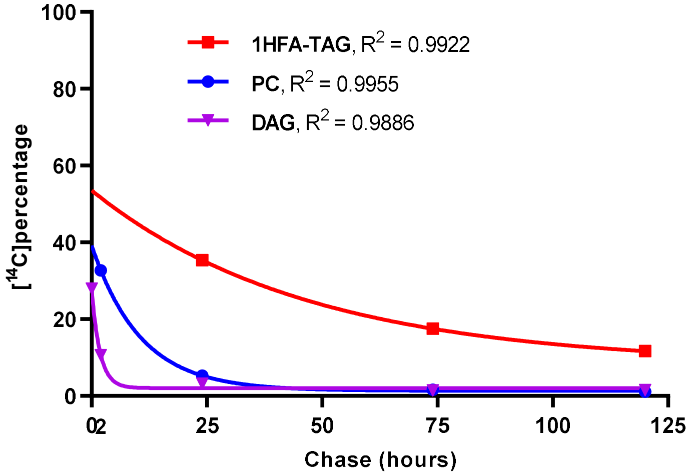

Supplemental Figure S6. Fitting of [^{14}C]glycerol pulse-chase labeling data (Figure 6D) to one phase exponential decay curves. Each curve is fitted to the time points after the lipid pool has reached maximal labeling. Data are mean ± SEM of 3 individual labeling replicates of ten embryos for each time point replicate. The goodness of fit is indicated in the legend. The calculated half-life fore each lipid is: 1HFA-TAG, 32.5 hours; PC, 7.3 hours; 0HFA-DAG, 1.3 hours.

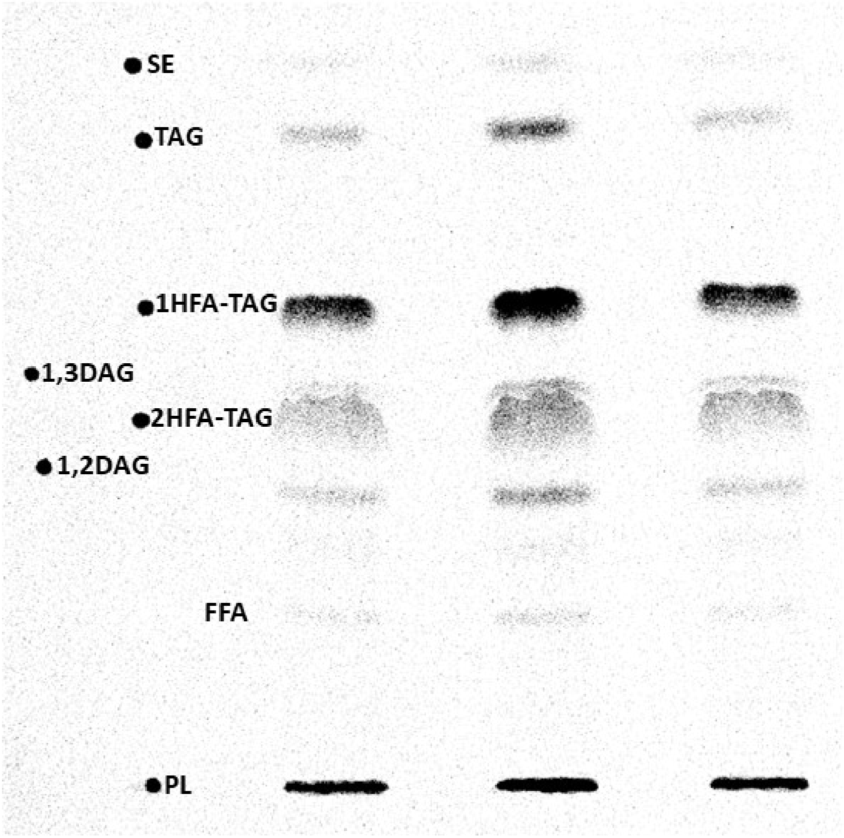

Supplemental Figure S7. Representative phosphor image for total lipid separation. Total lipid from 0 hr time point of [^{14}C]acetate pulse chase labeling experiment separated using dual HFA separation (Bates et al., 2011).

	Lipid % of total 14C (Fig. 4)				% 14C-FA in each lipid (Fig. 5)				% of total 14C composed of select FA				PUFA/20:1 in TAGs as % of total 14C
**Chase time**	**PC**	**0HFA-TAG**	**1HFA-TAG**	**2HFA-TAG**	**PC**	**0HFA-TAG**	**1HFA-TAG**	**2HFA-TAG**	**PC**	**0HFA-TAG**	**1HFA-TAG**	**2HFA-TAG**	
**24 hrs**	1.18	4.68	27.98	55.77	**% 18:1**				**% 18:1**				
					22.90	49.87	25.66	12.45	0.27	2.33	7.18	6.94	
					**% 18:2**				**% 18:2**				
					38.02	12.05	7.51	3.09	0.45	0.56	2.10	1.72	
					**% 18:3**				**% 18:3**				
					12.61	2.72	7.09	1.49	0.15	0.13	1.98	0.83	
									**Total PUFA**				
									0.60	0.69	4.09	2.55	7.33
					**% 20:1**				**% 20:1**				
					n/a	9.02	3.56	n/a	n/a	0.42	1.00	n/a	1.42
**168 hrs**	n/a	3.29	12.52	74.23	**% 18:2**				**% 18:2**				
					n/a	15.89	6.33	4.19	n/a	0.52	0.79	3.11	
					**% 18:3**				**% 18:3**				
					n/a	15.37	11.86	4.61	n/a	0.51	1.48	3.42	
									**Total PUFA**				
									n/a	1.03	2.28	6.53	9.84
					**% 20:1**				**% 20:1**				
					n/a	16.46	10.86	n/a	n/a	0.54	1.36	n/a	1.90

Supplemental Table S1. Increase in polyunsaturated fatty acids and 20:1 during [^{14}C]acetate chase 24-168 hrs as compared to substrates available in PC. The percent of total ^{14}C fatty acids as specific fatty acids in individual lipid species is calculated by multiplying lipid class ^{14}C by the percent of each labeled fatty acid in that lipid. 18:1 and total PUFA substrate available in PC at 24 hrs is highlighted in green. Yellow highlight is total PUFA in TAG species, orange highlight is total 20:1 in TAG species.

Gene Annotation	*Arabidopsis thaliana* TAIR ID	Gene Abbreviation for *Arabidopsis thaliana*	Gene Abbreviation for *Physaria fendleri*
Acyl-CoA : Diacylglycerol Acyltransferase 1	AT2G19450	AtDGAT1	PfeDGAT1
Acyl-CoA : Diacylglycerol Acyltransferase 2	AT3G51520	AtDGAT2	PfeDGAT2
Phosphatidylcholine:diacylglycerol cholinephosphotransferase	AT3G15820	AtPDCT/AtROD1	PfePDCT
Triacylglycerol Lipase like 1	AT1G23330	AtTAGL-1	PfeTAGL-like-1
Sugar-Dependent1 Lipase	AT5G04040	AtSDP1	PfeSDP1
Phospholipid : Diacylglycerol Acyltransferase 2	At3g44830	AtPDAT2	PfePDAT2

Supplemental Table S2. Potential *P. fendleri* genes involved in TAG remodeling. Gene abbreviations are mentioned in reference to Horn et al., 2016.
